# Synthesis, crystal structure and Hirshfeld analysis of *trans*-bis­(2-{1-[(6*R*,*S*)-3,5,5,6,8,8-hexa­methyl-5,6,7,8-tetra­hydronaphthalen-2-yl]ethyl­idene}-*N*-methyl­hydrazinecarbo­thio­amidato-κ^2^ *N* ^2^,*S*)palladium(II) ethanol monosolvate

**DOI:** 10.1107/S2056989023009908

**Published:** 2023-11-16

**Authors:** Ana Paula Lopes de Melo, Leandro Bresolin, Bárbara Tirloni, Renan Lira de Farias, Adriano Bof de Oliveira

**Affiliations:** a Escola de Química e Alimentos, Universidade Federal do Rio Grande, Av. Itália km 08, Campus Carreiros, 96203-900 Rio Grande-RS, Brazil; b Departamento de Química, Universidade Federal de Santa Maria, Av. Roraima 1000, Campus Universitário, 97105-900 Santa Maria-RS, Brazil; c Departamento de Química, Pontífícia Universidade Católica do Rio de Janeiro, Rua Marquês de São Vicente 225, 22451-900 Rio de Janeiro-RJ, Brazil; d Departamento de Química, Universidade Federal de Sergipe, Av. Marcelo Deda Chagas s/n, Campus Universitário, 49107-230 São Cristóvão-SE, Brazil; Venezuelan Institute of Scientific Research, Venezuela

**Keywords:** palladium(II) thio­semicarbazone-complex, fixolide 4-methyl­thio­semicarbazone, Hirshfeld surface analysis, anagostic inter­action, hydrogen-bonded macrocyclic environment, hydrogen-bonded ribbons, crystal structure

## Abstract

The reaction between the (*R*,*S*)-fixolide 4-methyl­thio­semicarbazone and Pd^II^ chloride yielded the title compound, [Pd(C_20_H_30_N_3_S)_2_]·C_2_H_6_O {common name: *trans*-bis­[(*R*,*S*)-fixolide 4-methyl­thio­semicarbazonato-κ^2^ *N* ^2^ *S*]palladium(II) ethanol monosolvate}. The asymmetric unit of the title compound consists of one bis-thio­semicarbazonato Pd^II^ complex and one ethanol solvent mol­ecule. The thio­semicarbazononato ligands act as metal chelators with a *trans* configuration in a distorted square-planar geometry. A C—H⋯S intra­molecular inter­action, with graph-set motif *S*(6), is observed and the coordination sphere resembles a hydrogen-bonded macrocyclic environment. Additionally, one C—H⋯Pd anagostic inter­action can be suggested. Each ligand is disordered over the aliphatic ring, which adopts a half-chair conformation, and two methyl groups [s.o.f. = 0.624 (2):0.376 (2)]. The disorder includes the chiral carbon atoms and, remarkably, one ligand has the (*R*)-isomer with the highest s.o.f. value atoms, while the other one shows the opposite, the atoms with the highest s.o.f. value are associated with the (*S*)-isomer. The N—N—C(=S)—N fragments of the ligands are approximately planar, with the maximum deviations from the mean plane through the selected atoms being 0.0567 (1) and −0.0307 (8) Å (r.m.s.d. = 0.0403 and 0.0269 Å) and the dihedral angle with the respective aromatic rings amount to 46.68 (5) and 50.66 (4)°. In the crystal, the complexes are linked *via* pairs of N—H⋯S inter­actions, with graph-set motif *R*_2_^2^(8), into centrosymmetric dimers. The dimers are further connected by centrosymmetric pairs of ethanol mol­ecules, building mono-periodic hydrogen-bonded ribbons along [011]. The Hirshfeld surface analysis indicates that the major contributions for the crystal cohesion are [atoms with highest/lowest s.o.f.s considered separately]: H⋯H (81.6/82.0%), H⋯C/C⋯H (6.5/6.4%), H⋯N/N⋯H (5.2/5.0%) and H⋯S/S⋯H (5.0/4.9%).

## Abstract 2

The synthesis, crystal structure and Hirshfeld analysis of the first complex with the (*R*,*S*)-fixolide 4-methyl­thio­semicarbazonato ligand is reported. A hydrogen-bonded macrocyclic environment type is observed for the Pd^II^ homoleptic complex. In the crystal, the complexes and the ethanol solvate mol­ecules are linked by H⋯S and H⋯O inter­actions, forming mono-periodic hydrogen-bonded ribbons along [011].

## 1. Chemical context

One of the first reports concerning thio­semicarbazone chemistry was published more than a century ago (Freund & Schander, 1902 ▸). These mol­ecules, with the [*R*_1_ *R*_2_N—N(H)—C(=S)—N*R*_3_ *R*_4_] functional group, were observed as the major product of the reactions between thio­semicarbazide derivatives [H_2_N—N(H)—C(=S)—N*R*_3_ *R*_4_] and aldehydes or ketones (*R*_1_ *R*_2_C=O). Indeed, thio­semicarbazides were employed as analytical reagents in the organic chemistry for the detection of the carbonyl group (*R*_1_ *R*_2_C=O). From those early times, thio­semicarbazones emerged as a class of compounds with applications in a wide range of scientific disciplines. A milestone of this chemistry was the report of the biological activity as chemotherapeutic agents against tuberculosis in *in vitro* essays, published in the mid-1940s (Domagk *et al.*, 1946 ▸).

As a result of the huge structural diversity of thio­semicarbazone derivatives, because of the large number of aldehydes and ketones employed in synthesis, several applications for metals, *e.g.*, palladium(II) are observed. The [N—N(H)—C(=S)—N] fragment, and its anionic form, are very efficient ligands, since hard (N) and soft (S) Lewis-base behaviors are present in the same atom chain. In addition, the N—N—S—N entity can adopt different geometries, coordinating metal centers in diverse bonding modes (Lobana *et al.*, 2009 ▸).

The applications of thio­semicarbazone derivatives in palladium chemistry range from analytical chemistry, *e.g.*, the spectrophotometric determination of Pd^II^ in different matrices, as for example alloys and complexes (Karthikeyan *et al.*, 2011 ▸), to their use as reagents for the synthesis of palladium nanoparticles for Suzuki–Miyaura cross-coupling catalysis (Kovala-Demertzi *et al.*, 2008 ▸) and the synergetic effect of thio­semicarbazones with palladium(II) has led to the development of catalysts for organic chemistry (Priyarega *et al.*, 2022 ▸). Furthermore, in the field of materials science, a palladium(II) coordination compound, with the 4-{bis­[4-(*p*-meth­oxy­phen­yl)thio­semicarbazone]}-2,3-butane derivative, has found application in electrocatalytic hydrogen production (Straistari *et al.*, 2018 ▸), which is an important topic for energy research today. Finally, bioinorganic chemistry is one of the most relevant approaches for thio­semicarbazone chemistry (Aly *et al.*, 2023 ▸; Singh *et al.*, 2023 ▸). 
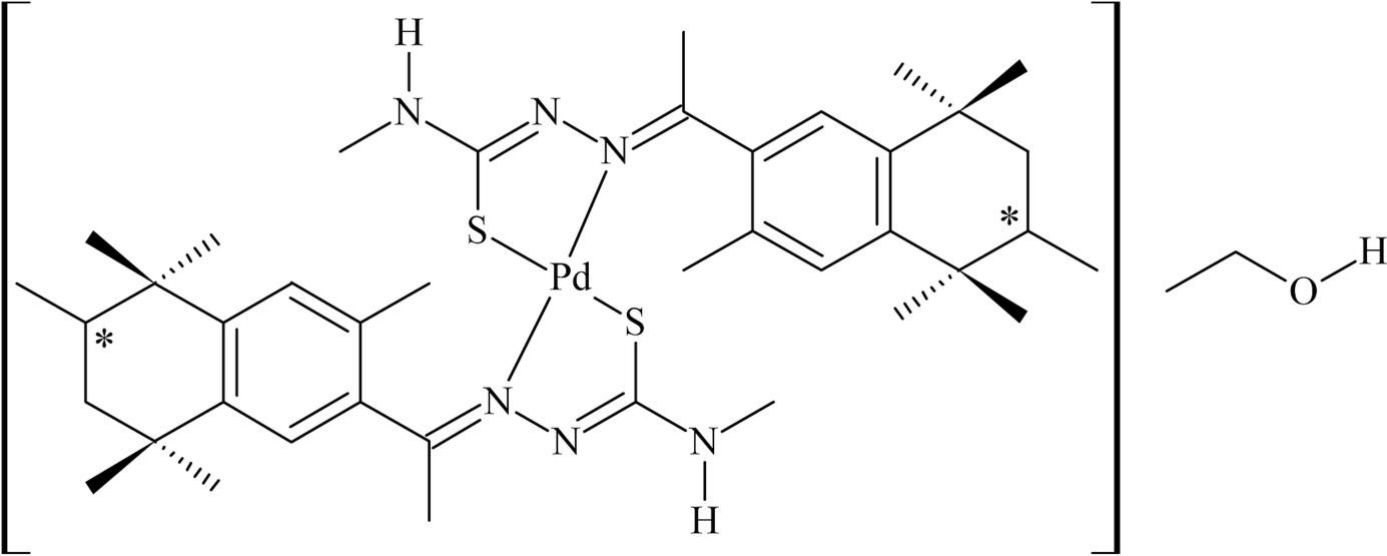


Herein, as part of our inter­est in thio­semicarbazone chemistry, we report the synthesis, crystal structure and Hirshfeld analysis of the first fixolide 4-methyl­thio­semicarbazonato palladium(II) complex.

## 2. Structural commentary

**Figure 1:**
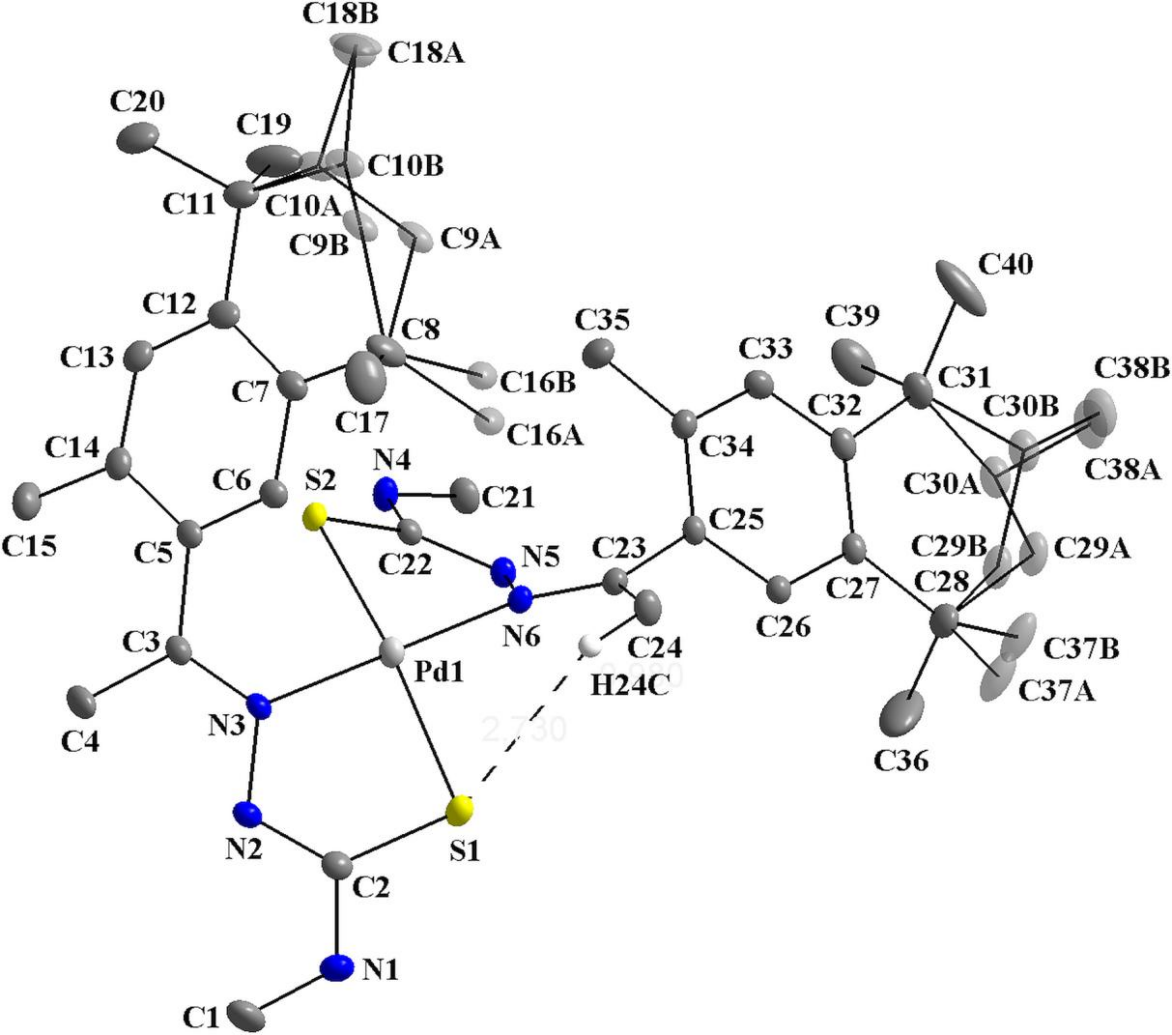
The mol­ecular structure of the title compound, showing the atom labeling and displacement ellipsoids drawn at the 40% probability level. Disordered atoms are drawn with 40% transparency and *A*-labeled for the (*R*)-isomer [s.o.f. = 0.624 (2)] and *B*-labeled for the (*S*)-isomer [s.o.f. = 0.376 (2)]. The ethanol solvate mol­ecule is omitted for clarity.

The asymmetric unit of the title compound consists of one bis-thio­semicarbazonato Pd^II^ complex and one ethanol solvate mol­ecule. The coordination compound is composed of a palladium(II) center and two (*R,S*)-fixolide 4-methyl­thio­semicarbazonato ligands, which act as metal chelators, κ^2^ *N* ^2^,*S*-donors, and form five-membered metallarings in a *trans*-configuration. An intra­molecular C24—H24*C*⋯S1 hydrogen bond is observed, with a graph-set motif of *S*(6), and the coordination sphere of the metal center resembles a hydrogen-bonded macrocyclic environment (Fig. 1 ▸, Table 1 ▸). The Pd^II^ metal center is fourfold coordinated in a distorted square-planar geometry: the N3—Pd1—N6 and S1—Pd1—S2 angles are 178.02 (5) and 164.63 (2)°, while the maximum deviation from the mean plane through the Pd1/N3/N6/S1/S2 atoms amounts to 0.1722 (4) Å for S1 [the r.m.s.d. for the selected atoms is 0.1409 Å] and the torsion angles between the N3—N2—C2—S1 and N6—N5—C22—S2 chains amount to −5.6 (2) and −1.7 (2)°. Additional structural data concerning the N/N/C/S/N entities are given in Table 2 ▸.

**Figure 2:**
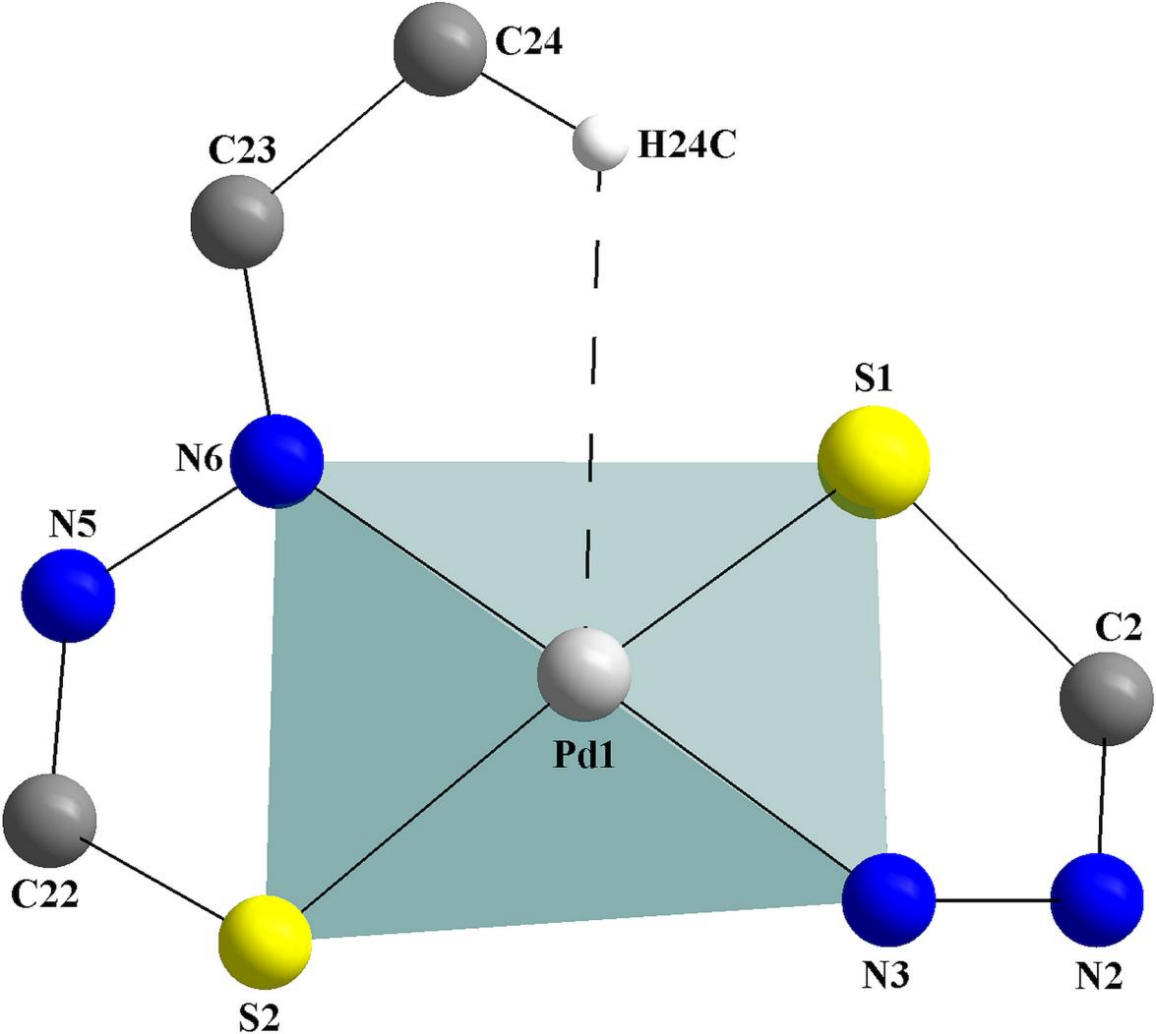
Graphical representation of the coordination sphere of the title compound showing the H⋯Pd weak anagostic intra­molecular inter­action. The figure is simplified for clarity.

In addition, a C24—H24*C*⋯Pd1 weak anagostic inter­action can be suggested (Fig. 2 ▸). The angle between the C—H⋯*M* atoms is 117.78 (2)° and the H⋯Pd distance amounts to 2.8235 (7) Å, which lies in the upper limit for these inter­actions. For an agostic inter­action, which involves a covalent or a three-center and two-electron bond, an H⋯*M* distance of at least 2.3 Å is required and the C—H⋯*M* angle should range between 90 and 140°. For an anagostic inter­action that is assigned with an electrostatic nature, the H⋯*M* distance should range from 2.3 to 2.9 Å and the C—H⋯*M* angle between 110 and 170° (Brookhart *et al.*, 2007 ▸). For an article that corroborates with the H24*C*⋯Pd1 anagostic inter­action of the title compound, see also: Derry Holaday *et al.* (2014 ▸).

In the complex, the thio­semicarbazonato ligands are disordered over the aliphatic rings and two of the methyl groups [site-occupancy ratio = 0.624 (2):0.376 (2)], with the *A*-labeled atoms having the highest s.o.f. value and the *B*-labeled atoms, the lowest (Fig. 1 ▸). For both ligands, the disorder includes the carbon chiral atoms (C10 and C30) and thus, (*R*)- and (*S*)-isomers are observed. The C10*A*—H*A* and C10*B*—H*B* bonds are in opposite directions, and the (*R*)-isomer is assigned for the *A*-labeled atoms [s.o.f. = 0.624 (2)]. For the case of the C30*A*—H30*A* and C30*B*—H30*B* bonds, the (*R*)-isomer is assigned to the *B*-labeled atoms [s.o.f. = 0.376 (2)]. This inverted site-occupancy ratio for the (*R*,*S*)-isomery in the two ligands is a remarkable feature of the complex structure. Selected structural data parameters are provided in Tables 2 ▸ and 3 ▸.

Finally, the anionic form of the (*R*,*S*)-fixolide 4-methyl­thio­semicarbazonato ligands is confirmed by the absence of the H acidic hydrazinic atom and by the changes on the bond lengths over the N—N—C—S fragment. In a neutral, non-coordinated, thio­semicarbazone derivative, N—N(H)—C=S entity, the H hydrazinic atom is present, the N—N and N—C distances are characteristic for single bonds, while the C=S distance indicates a double bond. When the thio­semicarbazone is deprotonated with a base, *e.g.* NaOH, the negative charge is delocalized over the N—N—C—S chain and the values for the chemical bonds distances tend to inter­mediate lengths. Thus, the N—N bond length tends to be longer, maintaining single-bond character, the N—C bond lengths tend to be shorter, suggesting a double-bond character and the C—S bond lengths tend to be longer, indicating a single-bond character (Table 4 ▸).

## 3. Supra­molecular features

**Figure 3:**
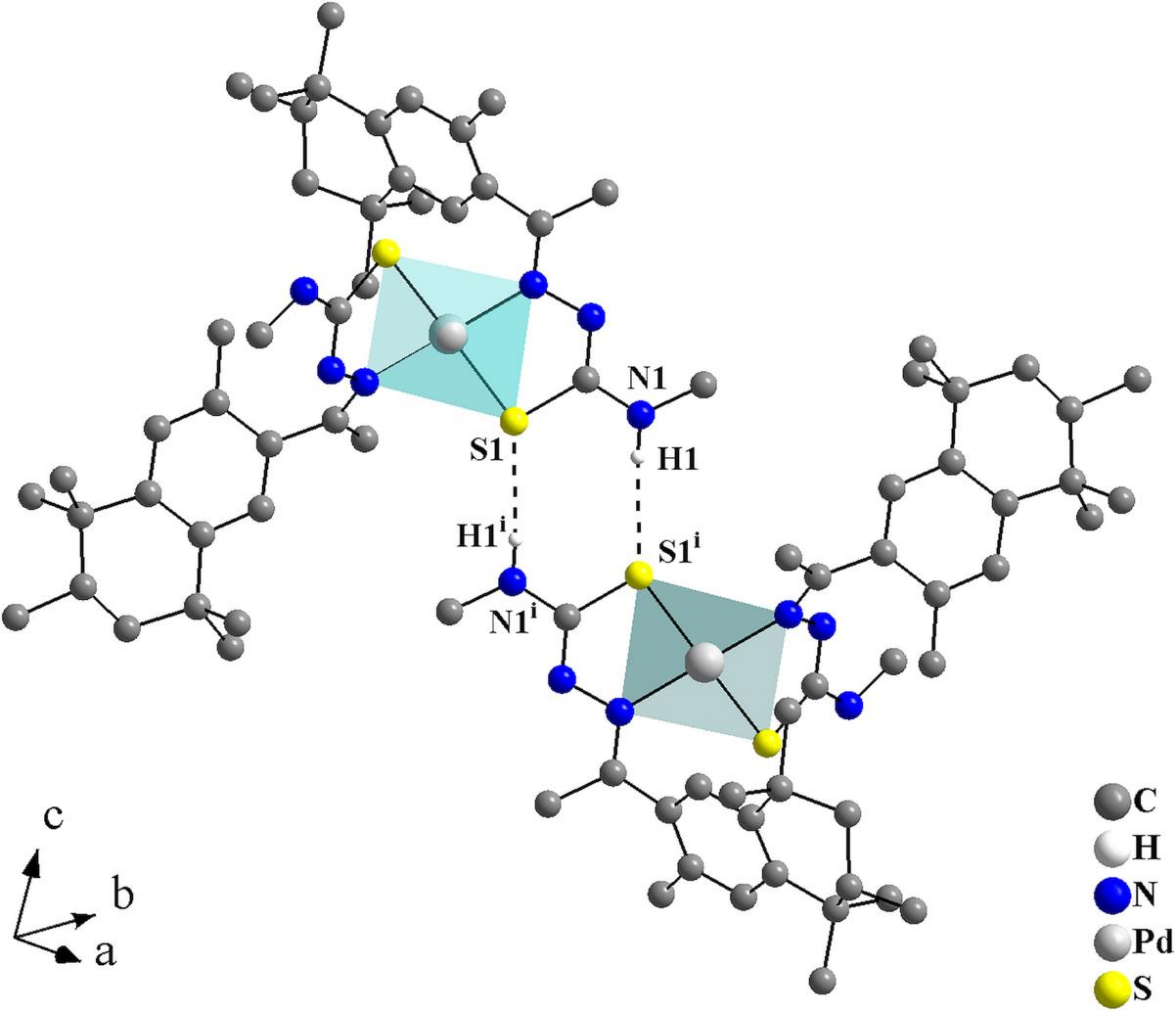
Graphical representation of the H⋯S inter­molecular inter­actions for the complex of the title compound, forming a graph-set motif of 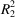 (8) and linking the mol­ecules into centrosymmetric dimers. The solvate mol­ecule is omitted and the figure is simplified for clarity [Symmetry code: (i) −*x* + 2, −*y* + 2, −*z* + 1.]

**Figure 4:**
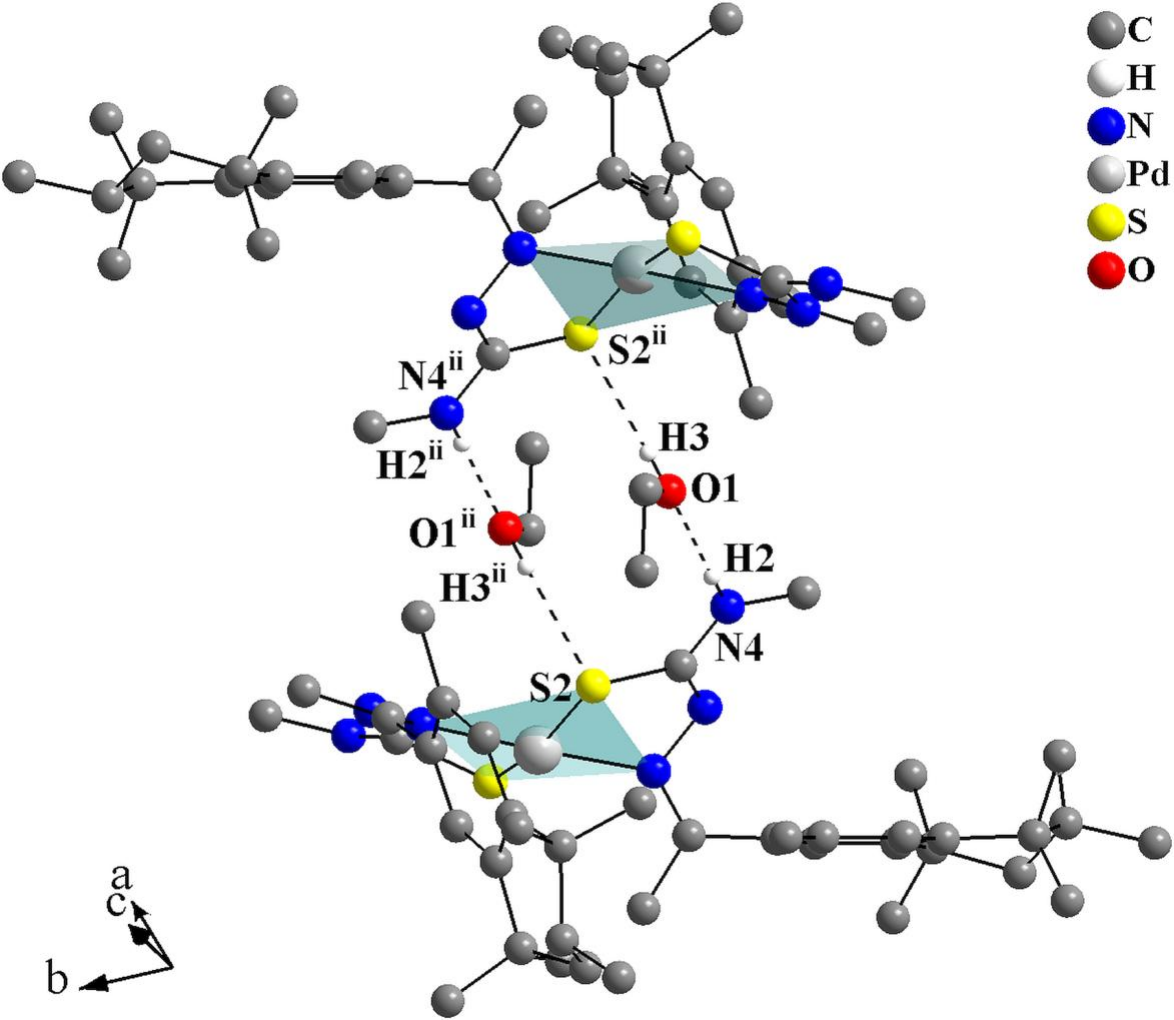
Graphical representation of the H⋯O and H⋯S inter­molecular inter­actions for the title compound drawn as dashed lines. Two ethanol solvate mol­ecules act as bridges connecting two complexes into centrosymmetric dimers. The figure is simplified for clarity. [Symmetry code: (ii) −*x* + 2, −*y* + 1, −*z* + 2.]

**Figure 5:**
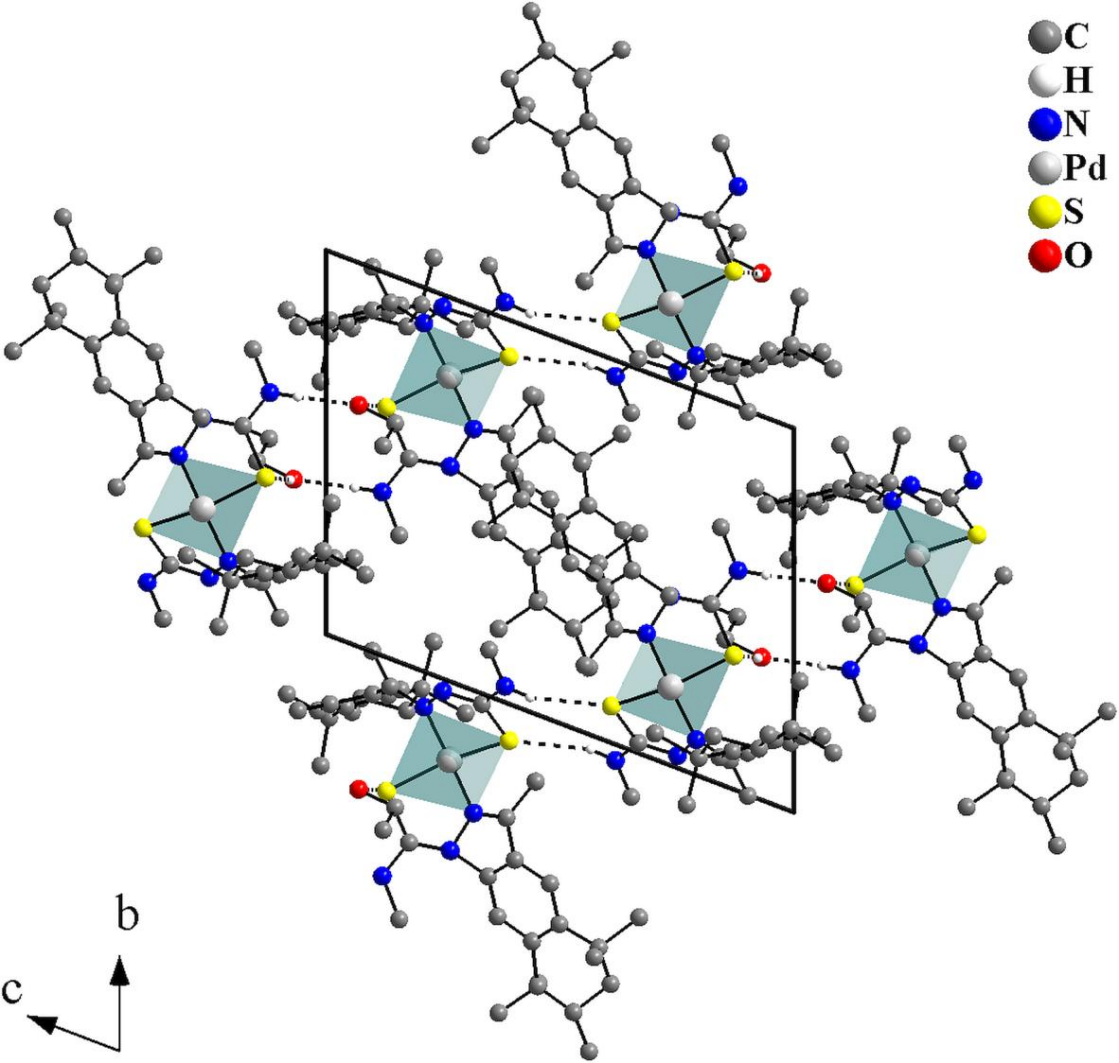
Crystal structure section of the title compound viewed along [100]. The H⋯O and H⋯S inter­molecular inter­actions are drawn as dashed lines and link the mol­ecules into mono-periodic hydrogen-bonded ribbons along [011].

In the crystal, the coordination compounds are connected through N—H⋯S inter­actions into centrosymmetric dimers with graph-set 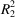 (8) (Fig. 3 ▸, Table 1 ▸). These dimers can be considered subunits of a hydrogen-bonded ribbon, since they are further linked by centrosymmetric pairs of ethanol solvate mol­ecules through N—H⋯O—H⋯S bridges (Fig. 4 ▸) into mono-periodic hydrogen-bonded ribbons along [011] (Fig. 5 ▸). The O1 atoms serve as hydrogen-atom acceptors and donors and the S1 atoms act as bifurcated hydrogen-atom acceptors.

**Figure 6:**
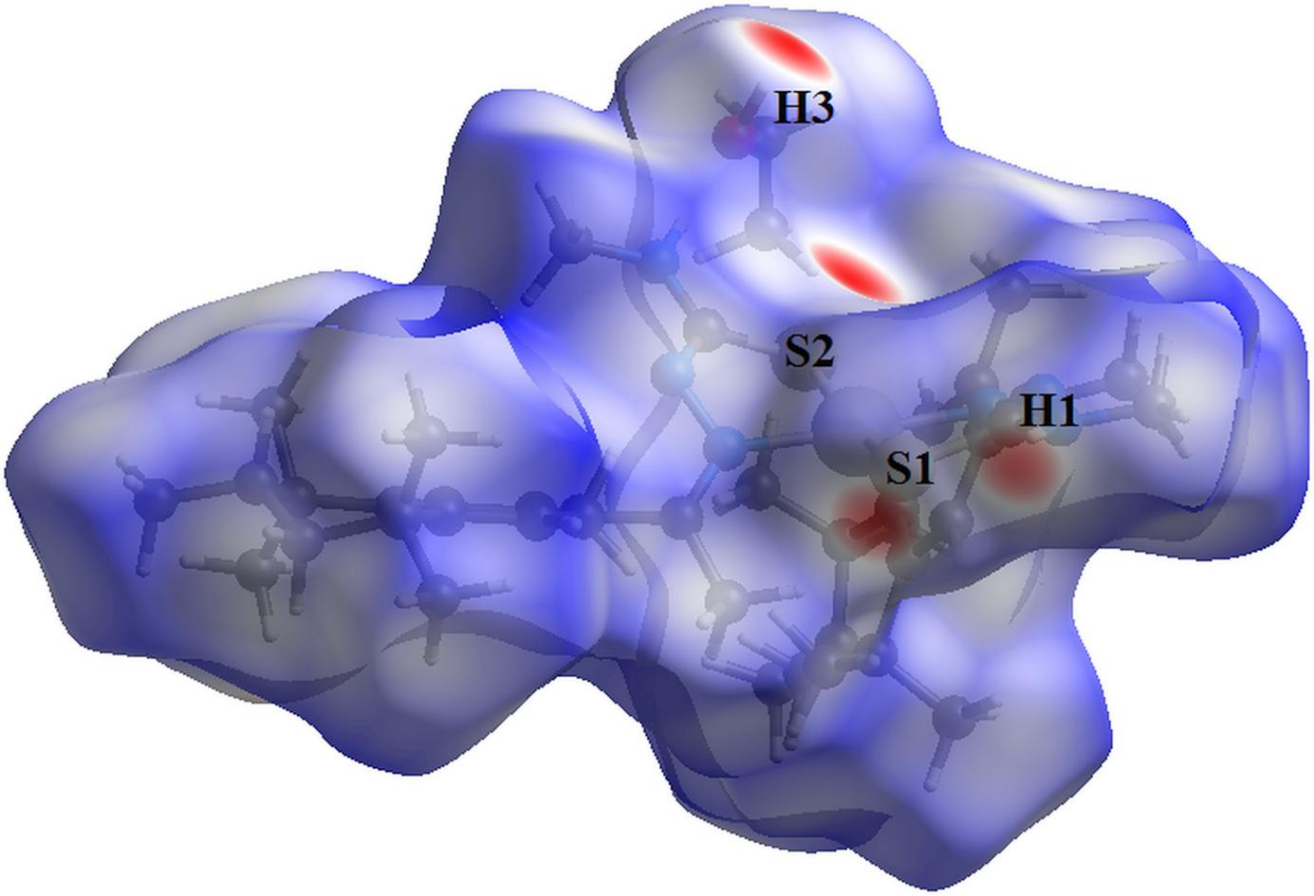
Hirshfeld surface graphical representation (*d*_norm_) for the title compound. The surface is drawn with transparency and the regions with strongest inter­molecular inter­actions are shown in magenta. [*d*_norm_ range: −0.427 to 1.632]

**Figure 7:**
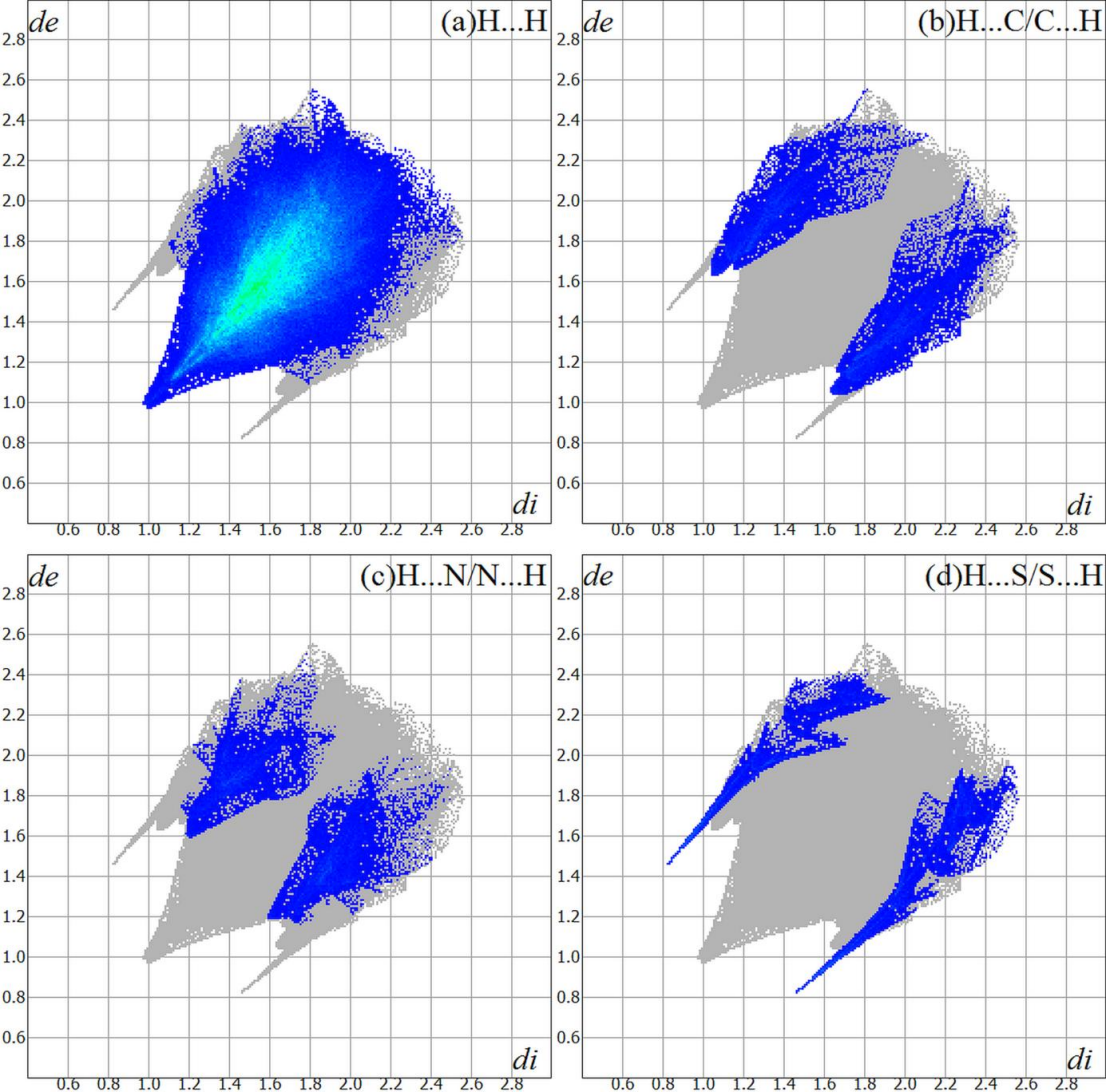
The Hirshfeld surface two-dimensional fingerprint plot for the title compound showing the inter­molecular contacts in detail (cyan dots). The major contributions to the crystal cohesion amount to (*a*) H⋯H = 81.6%, (*b*) H⋯C/C⋯H = 6.5%, (*c*) H⋯N/N⋯H = 5.2% and (*d*) H ⋯S/S⋯H = 5.0%. The *d*_i_ (*x*-axis) and the *d*_e_ (*y*-axis) values are the closest inter­nal and external distances from given points on the Hirshfeld surface (in Å).

For the title compound, the Hirshfeld surface analysis (Hirshfeld, 1977 ▸), the graphical representations and the two-dimensional Hirshfeld surface fingerprint were performed with *Crystal Explorer* software (Wolff *et al.*, 2012 ▸). The Hirshfeld surface analysis of the (*R*)-isomer structure of the title compound indicates that the most relevant inter­molecular inter­actions for crystal cohesion are the following: H⋯H (81.6%), H⋯C/C⋯H (6.5%), H⋯N/N⋯H (5.2%) and H⋯S/S⋯H (5.0%). Just for comparison, the (*S*)-isomer values amount to H⋯H (82.0%), H⋯C/C⋯H (6.4%), H⋯N/N⋯H (5.0%) and H⋯S/S⋯H (4.9%) and are quite similar to the results for the (*R*)-isomer. Since no considerable differences between the isomers was observed, the further evaluations and graphics were performed for the (*R*)-isomer only, which has the highest s.o.f. value. The graphical representations of the Hirshfeld surface for the *trans*-bis­[(*R*,*S*)-fixolide 4-methyl­thio­semicarbazonato-κ^2^ *N* ^2^ *S*]palladium(II) and the ethanol solvate mol­ecule are represented with transparency and using the ball-and-stick model (Fig. 6 ▸). The locations of the strongest inter­molecular contacts, *i.e*, the regions around the H1, H3, S1 and S2 atoms are indicated in magenta. These atoms are those involved in the H⋯S inter­actions shown in previous figures (Figs. 3 ▸, 4 ▸ and 5 ▸). The contributions to the crystal packing are shown as two-dimensional Hirshfeld surface fingerprint plots (HSFP) with cyan dots (Fig. 7 ▸). The *d*_i_ (*x-*axis) and the *d*_e_ (*y-*axis) values are the closest inter­nal and external distances from given points on the Hirshfeld surface contacts (in Å).

## 4. Database survey

To the best of our knowledge and from using database tools such as *SciFinder* (Chemical Abstracts Service, 2023 ▸) and the Cambridge Structural Database (CSD, accessed *via* WebCSD on October 21, 2023; Groom *et al.*, 2016 ▸), this work is the first attempt at the synthesis, crystal structure and Hirshfeld analysis of a (*R*,*S*)-fixolide-thio­semicarbazonato complex. Thus, three crystal structures with some similarities to the title compound were selected for comparison.

**Figure 8:**
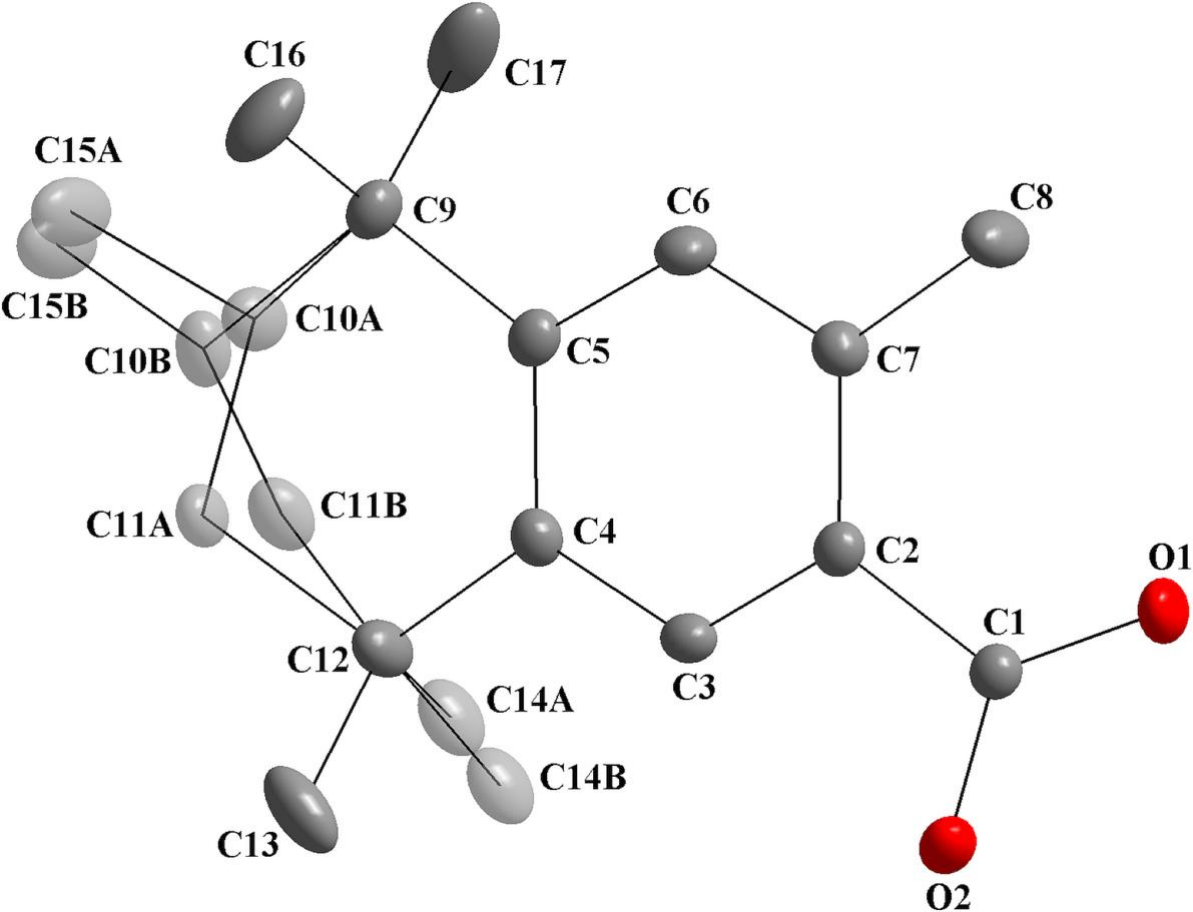
The mol­ecular structure of the (*R*,*S*)-fixolide carb­oxy­lic acid derivative, showing the atom labeling and displacement ellipsoids drawn at the 40% probability level (Kuhlich *et al.*, 2010 ▸). Disordered atoms are drawn with 40% transparency and *A*-labeled for the (*R*)-isomer [s.o.f. = 0.683 (4)] and *B*-labeled for the (*S*)-isomer [s.o.f. = 0.317 (4)].

The first selected compound is the (*R*,*S*)-fixolide carb­oxy­lic acid derivative (Kuhlich *et al.*, 2010 ▸). In this structure, only one crystallographically independent mol­ecule is observed in the asymmetric unit, which is disordered over the aliphatic ring and two methyl groups (Fig. 8 ▸). The chiral centers are disordered, C10*A* and C10*B*, so two isomers are observed, with *A*- and *B*-labeled atoms and related to the (*R*)- and (*S*)-isomers, as observed for the title compound (Table 4 ▸).

**Figure 9:**
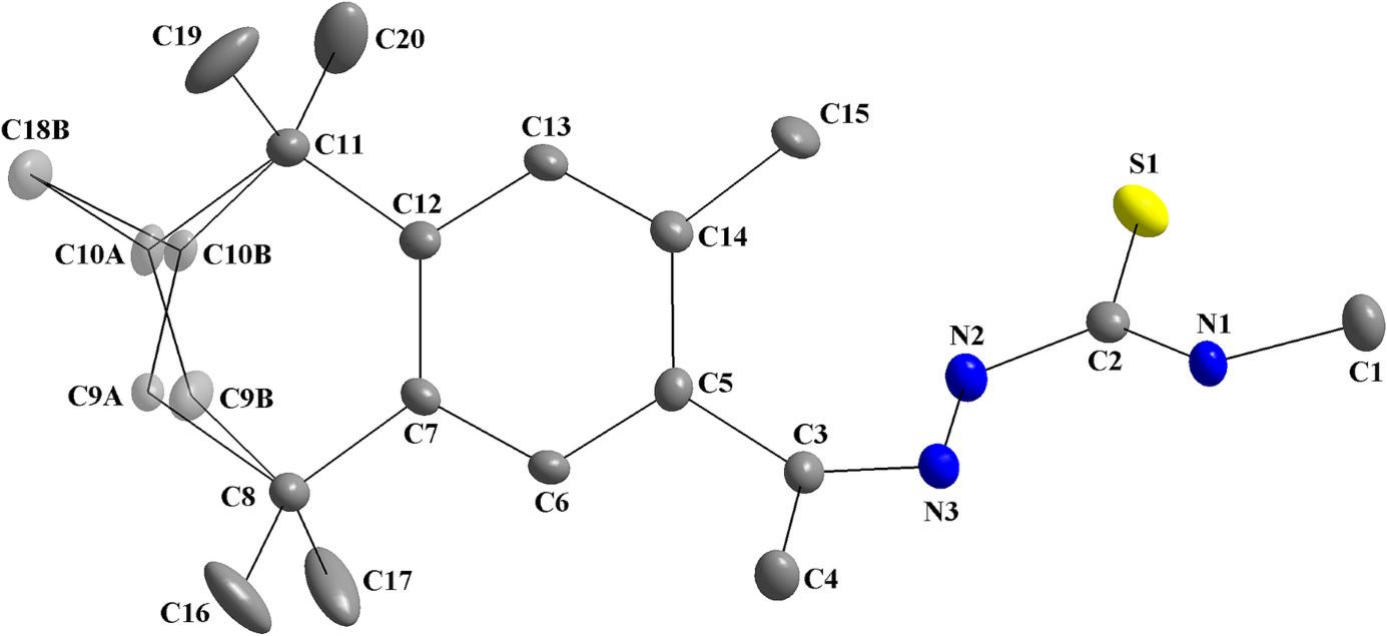
The mol­ecular structure of (*R*,*S*)-fixolide 4-methyl­thio­semicarbazone, showing the atom labeling and displacement ellipsoids drawn at the 40% probability level (Melo *et al.*, 2023*a* ▸). Disordered atoms are drawn with 40% transparency and *A*-labeled for the (*R*)-isomer [s.o.f. = 0.646 (14)] and *B*-labeled for the (*S*)-isomer [s.o.f. = 0.354 (14)].

The second selected mol­ecule for comparison is the (*R*,*S*)-fixolide 4-methyl­thio­semicarbazone ligand (Melo *et al.*, 2023*a* ▸), which is disordered over the fixolide group (Fig. 9 ▸) and was employed in the synthesis of the title compound. The structural similarities and differences between non-coordinated and coordinated mol­ecules are shown in Tables 4 ▸ and 5 ▸. For the (*R*,*S*)-fixolide 4-methyl­thio­semicarbazone, a distorted geometry is also observed, in particular between the aromatic ring and the thio­semicarbazone entity, with a dihedral angle of 51.77 (1)°.

**Figure 10:**
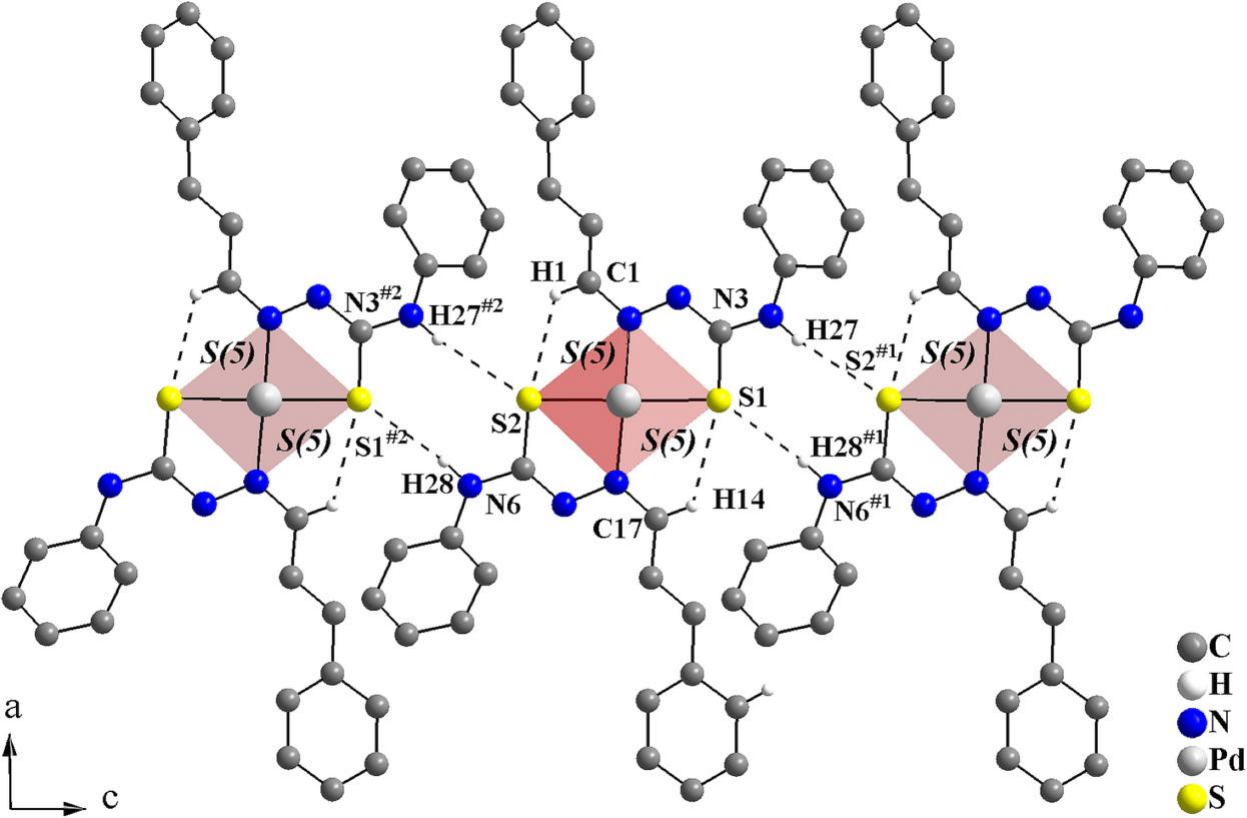
Crystal structure section of the *trans*-bis­[cinnamaldehyde 4-phenyl­thio­semicarbazonato-κ^2^ *N* ^2^ *S*]palladium(II) complex viewed along [010] (Melo *et al.*, 2023*b* ▸). The H⋯S intra- and inter­molecular hydrogen bonds are drawn as dashed lines, forming rings of graph-set motifs 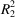 (8) and *S*(5). The mol­ecules are connected into mono-periodic hydrogen-bonded ribbons along [001]. [Symmetry codes: (#i) *x*, −*y* + 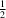 , *z* + 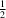 ; (#ii) *x*, −*y* + 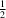 , *z* − 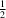 .]

Finally, a bis-thio­semicarbazonato Pd^II^ complex was chosen for comparison. In the crystal structure of the *trans*-bis­[cinnamaldehyde 4-phenyl­thio­semicarbazonato-κ^2^ *N* ^2^ *S*]palladium(II) compound (Melo *et al.*, 2023*b* ▸), the mol­ecules are also connected by N—H⋯S inter­molecular inter­actions, forming rings of graph-set motif 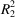 (8), and linked into mono-periodic hydrogen-bonded ribbons along [001]. In addition, C—H⋯S intra­molecular inter­actions are observed, with rings of graph-set motif *S*(5). Similar to the title compound, a hydrogen-bonded macrocyclic coordination environment can be suggested, with the sulfur atoms acting as bifurcated hydrogen-bond acceptors, *e.g.*, C17—H14⋯S1 and N6^#i^—H28^#1^⋯S1 [symmetry code: (#i) *x*, −*y* + 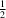 , *z* + 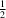 ] (Fig. 10 ▸).

## 5. Synthesis and crystallization

The starting materials are commercially available and were used without further purification. The synthesis of the complex was adapted from a previously reported procedure (Melo *et al.*, 2023*b* ▸). An ethano­lic solution of (*R*,*S*)-fixolide 4-methyl­semicarbazone (4 mmol, 50 mL) was prepared and the ligand was deprotonated with one pellet of NaOH. The solution was stirred for 4 h, until a yellow color could be observed. Simultaneously, an ethano­lic suspension of palladium(II) chloride (2 mmol, 50 mL) was prepared under stirring. A yellow-colored mixture of both ethano­lic solution and suspension was maintained with stirring at room temperature for 8 h, until the PdCl_2_ was consumed. Orange single crystals suitable for X-ray diffraction were obtained by the slow evaporation of the solvent.

## 6. Refinement

Crystal data, data collection and structure refinement details are summarized in Table 6 ▸. The crystallographically independent bis-thio­semicarbazonato Pd^II^ complex is disordered over the fixolide fragments (Fig. 1 ▸). Thus, the C9, C10, C16, C18, C29, C30, C37 and C38 atoms were split into two positions labeled *A* and *B*, with a refined site-occupancy ratio of 0.624 (2):0.376 (2). The EADP command was used to constrain the displacement parameters of the disordered atoms to get a stable refinement. Although the displacement ellipsoids of the C17, C19, C20, C36, C39 and C40 atoms seen to be prolate-like, no additional disorder was indicated by the data analysis.

Hydrogen atoms were located in difference-Fourier maps but were positioned with idealized geometry and refined isotropically using a riding model (HFIX command). Methyl H atoms were allowed to rotate but not to tip to best fit the experimental electron density. So, for the methyl H atoms, with [*U*_iso_(H) = 1.5*U*_eq_(C)], the C—H bond lengths were set to 0.98 Å. The other C—H bond lengths were also set according to the H-atom neighborhood, with [*U*_iso_(H) = 1.2*U*_eq_(C)]. For the phenyl H atoms, the C—H bond lengths were set to 0.95 Å, for the H atoms of the disordered –CH_2_– fragments (C9*A*, C9*B*, C29*A* and C29*B*), the C—H bond lengths were set to 0.99 Å and for the H atoms attached to the disordered tertiary C atoms (C10*A*, C10*B*, C30*A* and C30*B*), the C—H bond lengths were set to 1.00 Å. Finally, the N—H bond lengths, with [*U*_iso_(H) = 1.2*U*_eq_(N)], were set to 0.88 Å.

## Supplementary Material

Crystal structure: contains datablock(s) I, publication_text. DOI: 10.1107/S2056989023009908/zn2034sup1.cif

Structure factors: contains datablock(s) I. DOI: 10.1107/S2056989023009908/zn2034Isup2.hkl

CCDC reference: 2308228

Additional supporting information:  crystallographic information; 3D view; checkCIF report

## Figures and Tables

**Table 1:** Hydrogen-bond geometry (Å, °)

*D*—H⋯*A*	*D*—H	H⋯*A*	*D*⋯*A*	*D*—H⋯*A*
C24—H24*C*⋯S1	0.98	2.73	3.5061 (19)	136
N1—H1⋯S1^i^	0.88	2.57	3.411 (2)	160
N4—H2⋯O1	0.88	2.01	2.879 (2)	167
O1—H3⋯S2^ii^	0.84	2.43	3.2596 (16)	169

Symmetry codes: (i) 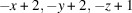 ; (ii) 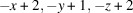 .

**Table 2:** The maximum deviations from the mean plane through the N/N/C/S/N entities, the r.m.s. deviations of the selected atoms and the dihedral angle with the respective aromatic rings for the title compound (Å, °) The dihedral angle between the N/N/C/S/N entities amounts to 61.34 (4)°.

N/N/C/S/N entity	max. deviation	Atom	r.m.s.d.	Angle
N3/N2/C2/N1/S1	0.0567 (1)	N2	0.0403	46.68 (5)
N6/N5/C22/N4/S2	−0.0307 (8)	N6	0.0269	50.66 (4)

**Table 3:** The maximum deviations from the mean plane through the aliphatic rings and the respective r.m.s. deviations of the selected atoms for the title compound (Å)

Aliphatic ring	max. deviation (+)	max. deviation (-)	r.m.s.d.
C7/C8/C9*A*/C10*A*/C11/C12	0.347 (2) (C10*A*)	−0.343 (2) (C9*A*)	0.2152
C7/C8/C9*B*/C10*B*/C11/C12	0.402 (3) (C9*B*)	−0.331 (3) [C10*B*]	0.2309
C27/C28/C29*A*/C30*A*/C31/C32	0.308 (2) (C30*A*)	−0.348 (2) [C29*A*]	0.2052
C27/C28/C29*B*/C30*B*/C31/C32	0.352 (4) (C29*B*)	−0.379 (4) (C30*B*)	0.2280

For a graphical representation of the title compound, see: Fig. 1 ▸.

**Table 4:** Selected torsion angles for disordered fixolide derivatives (°)

Compound	Isomer	Chiral atom (s.o.f.)	Atom chain	Torsion angle
C_17_H_24_O_2_^*a*^	*R*	C10*A* [0.683 (4)]	C9—C10*A*—C11*A*—C12	−67.0 (3)
C_17_H_24_O_2_^*a*^	*S*	C10*B* [0.317 (4)]	C9—C10*B*—C11*B*—C12	71.8 (6)
C_20_H_31_N_3_S^*b*^	*R*	C10*A* [0.646 (14)]	C8—C9*A*—C10*A*—C11	−66.4 (7)
C_20_H_31_N_3_S^*b*^	*S*	C10*B* [0.354 (14)]	C8—C9*B*—C10*B*—C11	67.7 (16)
Pd(C_20_H_30_N_3_S)_2_·C_2_H_6_O^*c*^	*R*	C10*A* [0.624 (2)]	C8—C9*A*—C10*A*—C11	−68.3 (3)
Pd(C_20_H_30_N_3_S)_2_·C_2_H_6_O^*c*^	*S*	C10*B* [0.376 (2)]	C8—C9*B*—C10*B*—C11	71.0 (4)
Pd(C_20_H_30_N_3_S)_2_·C_2_H_6_O^*c*^	*R*	C30*B* [0.3752 (2)]	C28—C29*B*—C30*B*—C31	−71.5 (5)
Pd(C_20_H_30_N_3_S)_2_·C_2_H_6_O^*c*^	*S*	C30*A* [0.624 (2)]	C28—C29*A*—C30*A*—C31	64.7 (3)

(*a*) The (*R*,*S*)-fixolide carb­oxy­lic acid derivative structure (Kuhlich *et al.*, 2010 ▸); (*b*) the (*R*,*S*)-fixolide 4-methyl­thio­semicarbazone structure (Melo *et al.*, 2023*a* ▸); (*c*) the *trans*-bis­[(*R*,*S*)-fixolide 4-methyl­thio­semicarbazonato-κ^2^ *N* ^2^ *S*]palladium(II) complex structure of this work.

**Table 5:** Bond lengths for the N—N—C—S entities in the neutral, non-coordinated, and the anionic, coordinated, forms of thio­semicarbazone derivatives (Å)

	N—N	N—C	C—S
C_20_H_31_N_3_S^*a*^	1.386 (3)	1.376 (4)	1.666 (3)
Pd(C_16_H_14_N_3_S)_2_^*b*^	1.390 (2)	1.293 (2)	1.7520 (19)
	1.393 (2)	1.291 (2)	1.7328 (19)
Pd(C_20_H_30_N_3_S)_2_·C_2_H_6_O^*c*^	1.3970 (18)	1.304 (2)	1.7520 (17)
	1.4056 (18)	1.306 (2)	1.7644 (16)

(*a*) The neutral and non-coordinated form of the (*R*,*S*)-fixolide 4-methyl­thio­semicarbazone structure (Melo *et al.*, 2023*a* ▸); (*b*) the anionic and coordinated form of the cinnamaldehyde 4-phenyl­thio­semicarbazone in a Pd^II^-complex (Melo *et al.*, 2023*b* ▸); (*c*) the anionic and coordinated form of the (*R*,*S*)-fixolide 4-methyl­thio­semicarbazone in the Pd^II^ complex of this work.

**Table 6:** Experimental details

Crystal data	
Chemical formula	[Pd(C_20_H_30_N_3_S)_2_]·C_2_H_6_O
*M*_r_	841.52
Crystal system, space group	Triclinic, *P* 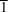
Temperature (K)	100
*a*, *b*, *c* (Å)	12.412 (3), 12.430 (4), 15.700 (5)
α, β, γ (°)	69.021 (5), 86.730 (6), 75.348 (9)
*V* (Å^3^)	2186.6 (11)
*Z*	2
Radiation type	Mo *K*α
μ (mm^−1^)	0.56
Crystal size (mm)	0.22 × 0.16 × 0.15

Data collection	
Diffractometer	Bruker D8 Venture Photon 100 diffractometer
Absorption correction	Multi-scan (*SADABS*; Krause *et al.*, 2015 ▸)
*T*_min_, *T*_max_	0.712, 0.746
No. of measured, independent and observed [*I* > 2σ(*I*)] reflections	102991, 10905, 10009
*R*_int_	0.033
(sin θ/λ)_max_ (Å^−1^)	0.668

Refinement	
*R*[*F* ^2^ > 2σ(*F* ^2^)], *wR*(*F* ^2^), *S*	0.028, 0.069, 1.04
No. of reflections	10905
No. of parameters	524
H-atom treatment	H-atom parameters constrained
Δρ_max_, Δρ_min_ (e Å^−3^)	1.16, −0.85

Computer programs: *APEX3* and *SAINT* (Bruker, 2015 ▸), *SHELXT2014/5* (Sheldrick, 2015*a* ▸), *SHELXL2018/3* (Sheldrick, 2015*b* ▸), *DIAMOND* (Brandenburg, 2006 ▸), CrystalExplorer (Wolff *et al.*, 2012 ▸), *WinGX* (Farrugia, 2012 ▸), *publCIF* (Westrip, 2010 ▸) and *enCIFer* (Allen *et al.*, 2004 ▸).

## supplementary crystallographic information

### Crystal data

**Table:**

[Pd(C_20_H_30_N_3_S)_2_]·C_2_H_6_O	*Z* = 2
*M**_r_* = 841.52	*F*(000) = 892
Triclinic, *P*1	*D*_x_ = 1.278 Mg m^−^^3^
*a* = 12.412 (3) Å	Mo *K*α radiation, λ = 0.71073 Å
*b* = 12.430 (4) Å	Cell parameters from 9602 reflections
*c* = 15.700 (5) Å	θ = 2.7–28.3°
α = 69.021 (5)°	µ = 0.56 mm^−^^1^
β = 86.730 (6)°	*T* = 100 K
γ = 75.348 (9)°	Block, orange
*V* = 2186.6 (11) Å^3^	0.22 × 0.16 × 0.15 mm

### Data collection

**Table:**

Bruker D8 Venture Photon 100 diffractometer	10009 reflections with *I* > 2σ(*I*)
Radiation source: microfocus X ray tube, Bruker D8 Venture	*R*_int_ = 0.033
φ and ω scans	θ_max_ = 28.3°, θ_min_ = 2.2°
Absorption correction: multi-scan (SADABS; Krause *et al.*, 2015)	*h* = −16→16
*T*_min_ = 0.712, *T*_max_ = 0.746	*k* = −16→16
102991 measured reflections	*l* = −20→20
10905 independent reflections	

### Refinement

**Table:**

Refinement on *F*^2^	Primary atom site location: structure-invariant direct methods
Least-squares matrix: full	Hydrogen site location: inferred from neighbouring sites
*R*[*F*^2^ > 2σ(*F*^2^)] = 0.028	H-atom parameters constrained
*wR*(*F*^2^) = 0.069	*w* = 1/[σ^2^(*F*_o_^2^) + (0.029*P*)^2^ + 2.3103*P*] where *P* = (*F*_o_^2^ + 2*F*_c_^2^)/3
*S* = 1.03	(Δ/σ)_max_ = 0.004
10905 reflections	Δρ_max_ = 1.16 e Å^−^^3^
524 parameters	Δρ_min_ = −0.85 e Å^−^^3^
0 restraints	

### Special details

**Table:**

Geometry. All esds (except the esd in the dihedral angle between two l.s. planes) are estimated using the full covariance matrix. The cell esds are taken into account individually in the estimation of esds in distances, angles and torsion angles; correlations between esds in cell parameters are only used when they are defined by crystal symmetry. An approximate (isotropic) treatment of cell esds is used for estimating esds involving l.s. planes.

### Fractional atomic coordinates and isotropic or equivalent isotropic displacement parameters (Å^2^)

**Table:**

	*x*	*y*	*z*	*U*_iso_*/*U*_eq_	Occ. (<1)
C1	1.08585 (18)	1.1210 (2)	0.65159 (15)	0.0342 (4)	
H1A	1.127520	1.168275	0.604350	0.051*	
H1B	1.134183	1.074266	0.706296	0.051*	
H1C	1.021234	1.174275	0.666759	0.051*	
C2	0.98087 (13)	0.97343 (14)	0.66670 (11)	0.0185 (3)	
C3	0.84118 (13)	0.92795 (14)	0.86307 (11)	0.0166 (3)	
C4	0.88757 (15)	1.00236 (18)	0.90111 (13)	0.0262 (4)	
H4A	0.967967	0.968782	0.913298	0.039*	
H4B	0.851213	1.003077	0.958045	0.039*	
H4C	0.873863	1.083872	0.856853	0.039*	
C5	0.73904 (13)	0.89044 (14)	0.90042 (11)	0.0164 (3)	
C6	0.64685 (13)	0.93003 (14)	0.84064 (11)	0.0176 (3)	
H6A	0.653560	0.977882	0.778936	0.021*	
C7	0.54497 (13)	0.90243 (14)	0.86751 (12)	0.0184 (3)	
C8	0.44913 (15)	0.95257 (17)	0.79614 (14)	0.0285 (4)	
C9A	0.3614 (2)	0.8814 (3)	0.8273 (2)	0.0232 (5)	0.6242 (19)
H9A	0.294196	0.922087	0.786351	0.028*	0.6242 (19)
H9B	0.390797	0.801528	0.823371	0.028*	0.6242 (19)
C10A	0.3301 (2)	0.8685 (3)	0.9250 (2)	0.0231 (5)	0.6242 (19)
H10A	0.312912	0.949179	0.929860	0.028*	0.6242 (19)
C16A	0.4957 (3)	0.9324 (3)	0.7023 (2)	0.0240 (6)	0.6242 (19)
H16A	0.546387	0.983484	0.674675	0.036*	0.6242 (19)
H16B	0.432969	0.952692	0.659503	0.036*	0.6242 (19)
H16C	0.535779	0.848916	0.716313	0.036*	0.6242 (19)
C18A	0.2246 (14)	0.8232 (14)	0.9448 (10)	0.034 (2)	0.6242 (19)
H18A	0.238058	0.746874	0.935804	0.051*	0.6242 (19)
H18B	0.163640	0.881083	0.903282	0.051*	0.6242 (19)
H18C	0.204337	0.812422	1.008060	0.051*	0.6242 (19)
C29A	0.7483 (3)	0.3718 (3)	0.3920 (2)	0.0306 (6)	0.6242 (19)
H29A	0.671534	0.415459	0.367646	0.037*	0.6242 (19)
H29B	0.790596	0.349086	0.343042	0.037*	0.6242 (19)
C30A	0.7441 (3)	0.2612 (3)	0.4714 (2)	0.0272 (6)	0.6242 (19)
H30A	0.821275	0.221792	0.498735	0.033*	0.6242 (19)
C38A	0.7043 (10)	0.1745 (13)	0.4383 (10)	0.0364 (18)	0.6242 (19)
H38A	0.631401	0.214109	0.406461	0.055*	0.6242 (19)
H38B	0.698052	0.104253	0.490800	0.055*	0.6242 (19)
H38C	0.758145	0.150090	0.396418	0.055*	0.6242 (19)
C37A	0.7757 (5)	0.5732 (4)	0.3311 (3)	0.0407 (11)	0.6242 (19)
H37A	0.804504	0.556092	0.276628	0.061*	0.6242 (19)
H37B	0.811067	0.630880	0.339773	0.061*	0.6242 (19)
H37C	0.694898	0.606325	0.323277	0.061*	0.6242 (19)
C9B	0.3361 (4)	0.9418 (4)	0.8571 (4)	0.0232 (5)	0.3758 (19)
H9C	0.326305	0.993856	0.893638	0.028*	0.3758 (19)
H9D	0.269739	0.968867	0.815654	0.028*	0.3758 (19)
C10B	0.3459 (4)	0.8182 (5)	0.9183 (4)	0.0231 (5)	0.3758 (19)
H10B	0.374312	0.765932	0.881872	0.028*	0.3758 (19)
C16B	0.4618 (5)	0.9141 (6)	0.7263 (4)	0.0240 (6)	0.3758 (19)
H16D	0.527331	0.934174	0.692787	0.036*	0.3758 (19)
H16E	0.395415	0.952311	0.685464	0.036*	0.3758 (19)
H16F	0.472013	0.827662	0.749337	0.036*	0.3758 (19)
C18B	0.230 (2)	0.800 (2)	0.9518 (19)	0.041 (5)	0.3758 (19)
H18D	0.238512	0.719202	0.996540	0.062*	0.3758 (19)
H18E	0.182946	0.810887	0.899874	0.062*	0.3758 (19)
H18F	0.195277	0.857957	0.980398	0.062*	0.3758 (19)
C29B	0.7999 (5)	0.3255 (5)	0.4260 (4)	0.0306 (6)	0.3758 (19)
H29C	0.822719	0.313817	0.367850	0.037*	0.3758 (19)
H29D	0.854429	0.266951	0.474536	0.037*	0.3758 (19)
C30B	0.6846 (5)	0.3020 (5)	0.4484 (4)	0.0272 (6)	0.3758 (19)
H30B	0.626963	0.370707	0.407784	0.033*	0.3758 (19)
C38B	0.6815 (19)	0.188 (2)	0.4315 (18)	0.0364 (18)	0.3758 (19)
H38D	0.686099	0.202517	0.366004	0.055*	0.3758 (19)
H38E	0.611710	0.166367	0.453400	0.055*	0.3758 (19)
H38F	0.744722	0.123369	0.464385	0.055*	0.3758 (19)
C37B	0.7445 (10)	0.5437 (8)	0.3352 (6)	0.0407 (11)	0.3758 (19)
H37D	0.782626	0.531758	0.281691	0.061*	0.3758 (19)
H37E	0.743742	0.623012	0.334589	0.061*	0.3758 (19)
H37F	0.667820	0.536733	0.333748	0.061*	0.3758 (19)
C11	0.43150 (15)	0.78566 (17)	0.99371 (13)	0.0247 (4)	
C12	0.53601 (14)	0.83033 (14)	0.95798 (12)	0.0193 (3)	
C13	0.62871 (14)	0.79313 (15)	1.01780 (12)	0.0209 (3)	
H13A	0.622121	0.745404	1.079562	0.025*	
C14	0.72960 (14)	0.82199 (15)	0.99204 (11)	0.0190 (3)	
C15	0.82669 (15)	0.77370 (18)	1.06003 (12)	0.0268 (4)	
H15A	0.894541	0.744754	1.031379	0.040*	
H15B	0.812136	0.708020	1.112896	0.040*	
H15C	0.836488	0.836955	1.079996	0.040*	
C17	0.40989 (19)	1.08683 (19)	0.77040 (17)	0.0402 (5)	
H17A	0.383149	1.105594	0.824637	0.060*	
H17B	0.349237	1.118500	0.723970	0.060*	
H17C	0.472015	1.122903	0.746018	0.060*	
C19	0.4588 (2)	0.6525 (2)	1.01169 (19)	0.0452 (6)	
H19A	0.479150	0.638591	0.954586	0.068*	
H19B	0.393480	0.622029	1.035635	0.068*	
H19C	0.521284	0.611280	1.056403	0.068*	
C20	0.39893 (18)	0.8059 (2)	1.08336 (16)	0.0368 (5)	
H20A	0.458826	0.759193	1.129724	0.055*	
H20B	0.330178	0.780965	1.104131	0.055*	
H20C	0.386937	0.890557	1.073604	0.055*	
C21	0.91116 (15)	0.33047 (14)	0.84376 (12)	0.0217 (3)	
H21A	0.846101	0.333782	0.809464	0.033*	
H21B	0.931379	0.253779	0.894234	0.033*	
H21C	0.973861	0.339274	0.803166	0.033*	
C22	0.85251 (12)	0.53979 (13)	0.82282 (11)	0.0149 (3)	
C23	0.74272 (13)	0.70098 (14)	0.61482 (10)	0.0159 (3)	
C24	0.69026 (15)	0.82454 (15)	0.55224 (11)	0.0214 (3)	
H24A	0.609123	0.840594	0.557104	0.032*	
H24B	0.709552	0.832430	0.489245	0.032*	
H24C	0.717728	0.881609	0.569328	0.032*	
C25	0.71832 (13)	0.59952 (14)	0.59672 (10)	0.0156 (3)	
C26	0.76394 (13)	0.57287 (14)	0.52167 (11)	0.0170 (3)	
H26A	0.807084	0.622144	0.482175	0.020*	
C27	0.74890 (13)	0.47630 (14)	0.50186 (11)	0.0171 (3)	
C28	0.80284 (16)	0.45433 (17)	0.41750 (12)	0.0248 (4)	
C31	0.66562 (16)	0.29300 (15)	0.54837 (12)	0.0235 (3)	
C32	0.68504 (14)	0.40437 (14)	0.56004 (11)	0.0180 (3)	
C33	0.63708 (14)	0.43480 (15)	0.63406 (11)	0.0198 (3)	
H33A	0.592393	0.386892	0.672897	0.024*	
C34	0.65108 (14)	0.53056 (15)	0.65380 (11)	0.0183 (3)	
C35	0.59337 (16)	0.55897 (18)	0.73331 (13)	0.0273 (4)	
H35A	0.648308	0.537659	0.782790	0.041*	
H35B	0.537032	0.513437	0.755035	0.041*	
H35C	0.557336	0.644237	0.713677	0.041*	
C36	0.9289 (2)	0.4244 (3)	0.42744 (19)	0.0651 (9)	
H36A	0.954038	0.357862	0.484795	0.098*	
H36B	0.952135	0.493968	0.427852	0.098*	
H36C	0.962068	0.402101	0.376098	0.098*	
C39	0.71855 (18)	0.18481 (16)	0.63149 (16)	0.0359 (5)	
H39A	0.797822	0.180704	0.637269	0.054*	
H39B	0.710531	0.112296	0.623880	0.054*	
H39C	0.681228	0.192076	0.686596	0.054*	
C40	0.54081 (19)	0.30199 (19)	0.5461 (2)	0.0495 (7)	
H40A	0.508242	0.313965	0.601325	0.074*	
H40B	0.528820	0.228391	0.543184	0.074*	
H40C	0.505229	0.369401	0.492188	0.074*	
C41	0.76119 (17)	0.4532 (2)	1.12175 (14)	0.0331 (4)	
H41A	0.760807	0.534078	1.080670	0.050*	
H41B	0.721493	0.456491	1.176890	0.050*	
H41C	0.724160	0.415658	1.090845	0.050*	
C42	0.87835 (18)	0.3823 (3)	1.14735 (16)	0.0500 (7)	
H42A	0.916403	0.421569	1.176905	0.060*	
H42B	0.878660	0.302170	1.191799	0.060*	
N1	1.04828 (13)	1.04158 (14)	0.61810 (11)	0.0262 (3)	
H1	1.069987	1.037675	0.564706	0.031*	
N2	0.96365 (11)	0.96714 (12)	0.75090 (10)	0.0179 (3)	
N3	0.88191 (10)	0.90727 (11)	0.79084 (9)	0.0143 (2)	
N4	0.88465 (12)	0.42619 (12)	0.87994 (9)	0.0187 (3)	
H2	0.889638	0.409810	0.939159	0.022*	
N5	0.84996 (11)	0.55992 (11)	0.73529 (9)	0.0159 (3)	
N6	0.80513 (11)	0.68097 (11)	0.68467 (9)	0.0149 (2)	
O1	0.93682 (11)	0.37138 (14)	1.06952 (9)	0.0306 (3)	
H3	1.004560	0.366360	1.077320	0.046*	
Pd1	0.84134 (2)	0.79593 (2)	0.73601 (2)	0.01358 (4)	
S1	0.92010 (4)	0.90429 (4)	0.60956 (3)	0.02192 (9)	
S2	0.80874 (3)	0.65163 (3)	0.87072 (3)	0.01597 (8)	

### Atomic displacement parameters (Å^2^)

**Table:**

	*U*^11^	*U*^22^	*U*^33^	*U*^12^	*U*^13^	*U*^23^
C1	0.0345 (10)	0.0387 (11)	0.0355 (11)	−0.0246 (9)	0.0015 (8)	−0.0096 (9)
C2	0.0166 (7)	0.0160 (7)	0.0221 (8)	−0.0032 (6)	0.0000 (6)	−0.0066 (6)
C3	0.0159 (7)	0.0164 (7)	0.0206 (7)	−0.0031 (6)	−0.0008 (6)	−0.0105 (6)
C4	0.0242 (8)	0.0346 (10)	0.0332 (10)	−0.0128 (7)	0.0051 (7)	−0.0249 (8)
C5	0.0162 (7)	0.0162 (7)	0.0212 (8)	−0.0037 (6)	0.0019 (6)	−0.0124 (6)
C6	0.0189 (7)	0.0153 (7)	0.0210 (8)	−0.0047 (6)	0.0014 (6)	−0.0093 (6)
C7	0.0169 (7)	0.0148 (7)	0.0263 (8)	−0.0029 (6)	0.0006 (6)	−0.0111 (6)
C8	0.0201 (8)	0.0258 (9)	0.0359 (10)	−0.0086 (7)	−0.0064 (7)	−0.0036 (8)
C9A	0.0153 (10)	0.0217 (11)	0.0371 (14)	−0.0051 (9)	−0.0006 (9)	−0.0151 (10)
C10A	0.0179 (11)	0.0162 (15)	0.0425 (14)	−0.0049 (12)	0.0089 (10)	−0.0199 (13)
C16A	0.0196 (18)	0.0342 (15)	0.0219 (17)	−0.0114 (12)	0.0028 (11)	−0.0113 (14)
C18A	0.026 (3)	0.038 (4)	0.048 (4)	−0.014 (2)	0.011 (2)	−0.024 (3)
C29A	0.048 (2)	0.0266 (16)	0.0233 (15)	−0.0109 (13)	0.0006 (11)	−0.0150 (12)
C30A	0.0356 (16)	0.0214 (14)	0.0278 (14)	−0.0056 (11)	−0.0040 (11)	−0.0126 (11)
C38A	0.053 (5)	0.027 (3)	0.038 (2)	−0.011 (4)	−0.005 (3)	−0.019 (2)
C37A	0.076 (4)	0.030 (3)	0.0179 (11)	−0.0152 (16)	0.0111 (18)	−0.0105 (17)
C9B	0.0153 (10)	0.0217 (11)	0.0371 (14)	−0.0051 (9)	−0.0006 (9)	−0.0151 (10)
C10B	0.0179 (11)	0.0162 (15)	0.0425 (14)	−0.0049 (12)	0.0089 (10)	−0.0199 (13)
C16B	0.0196 (18)	0.0342 (15)	0.0219 (17)	−0.0114 (12)	0.0028 (11)	−0.0113 (14)
C18B	0.019 (6)	0.053 (11)	0.065 (9)	−0.021 (6)	0.020 (5)	−0.029 (7)
C29B	0.048 (2)	0.0266 (16)	0.0233 (15)	−0.0109 (13)	0.0006 (11)	−0.0150 (12)
C30B	0.0356 (16)	0.0214 (14)	0.0278 (14)	−0.0056 (11)	−0.0040 (11)	−0.0126 (11)
C38B	0.053 (5)	0.027 (3)	0.038 (2)	−0.011 (4)	−0.005 (3)	−0.019 (2)
C37B	0.076 (4)	0.030 (3)	0.0179 (11)	−0.0152 (16)	0.0111 (18)	−0.0105 (17)
C11	0.0225 (8)	0.0280 (9)	0.0300 (9)	−0.0106 (7)	0.0104 (7)	−0.0162 (7)
C12	0.0194 (7)	0.0170 (7)	0.0265 (8)	−0.0042 (6)	0.0074 (6)	−0.0145 (6)
C13	0.0244 (8)	0.0203 (8)	0.0200 (8)	−0.0042 (6)	0.0053 (6)	−0.0112 (6)
C14	0.0204 (8)	0.0197 (7)	0.0197 (8)	−0.0017 (6)	0.0009 (6)	−0.0125 (6)
C15	0.0248 (9)	0.0336 (10)	0.0220 (8)	−0.0017 (7)	−0.0026 (7)	−0.0130 (7)
C17	0.0394 (12)	0.0284 (10)	0.0467 (13)	0.0092 (9)	−0.0144 (10)	−0.0158 (9)
C19	0.0477 (13)	0.0335 (11)	0.0700 (17)	−0.0248 (10)	0.0332 (12)	−0.0315 (12)
C20	0.0367 (11)	0.0460 (12)	0.0415 (12)	−0.0197 (9)	0.0227 (9)	−0.0286 (10)
C21	0.0266 (8)	0.0145 (7)	0.0253 (8)	−0.0035 (6)	−0.0031 (7)	−0.0091 (6)
C22	0.0151 (7)	0.0143 (7)	0.0171 (7)	−0.0046 (5)	−0.0004 (5)	−0.0069 (6)
C23	0.0187 (7)	0.0157 (7)	0.0148 (7)	−0.0052 (6)	0.0018 (6)	−0.0068 (6)
C24	0.0276 (8)	0.0168 (7)	0.0190 (8)	−0.0042 (6)	−0.0051 (6)	−0.0055 (6)
C25	0.0181 (7)	0.0154 (7)	0.0147 (7)	−0.0042 (6)	−0.0016 (6)	−0.0065 (6)
C26	0.0191 (7)	0.0181 (7)	0.0155 (7)	−0.0064 (6)	0.0000 (6)	−0.0067 (6)
C27	0.0184 (7)	0.0169 (7)	0.0170 (7)	−0.0028 (6)	−0.0026 (6)	−0.0078 (6)
C28	0.0317 (9)	0.0268 (9)	0.0240 (9)	−0.0108 (7)	0.0065 (7)	−0.0170 (7)
C31	0.0313 (9)	0.0165 (8)	0.0255 (9)	−0.0085 (7)	−0.0032 (7)	−0.0087 (7)
C32	0.0209 (7)	0.0141 (7)	0.0185 (7)	−0.0032 (6)	−0.0055 (6)	−0.0052 (6)
C33	0.0225 (8)	0.0180 (7)	0.0191 (8)	−0.0085 (6)	−0.0003 (6)	−0.0043 (6)
C34	0.0206 (7)	0.0201 (8)	0.0156 (7)	−0.0060 (6)	0.0003 (6)	−0.0074 (6)
C35	0.0319 (9)	0.0343 (10)	0.0244 (9)	−0.0161 (8)	0.0108 (7)	−0.0168 (8)
C36	0.0327 (12)	0.124 (3)	0.0467 (15)	−0.0114 (15)	0.0162 (11)	−0.0482 (18)
C39	0.0350 (11)	0.0142 (8)	0.0528 (13)	0.0004 (7)	−0.0170 (9)	−0.0068 (8)
C40	0.0369 (12)	0.0223 (10)	0.090 (2)	−0.0030 (8)	−0.0347 (12)	−0.0182 (11)
C41	0.0319 (10)	0.0397 (11)	0.0323 (10)	−0.0041 (8)	−0.0010 (8)	−0.0209 (9)
C42	0.0278 (11)	0.095 (2)	0.0285 (11)	−0.0050 (12)	−0.0022 (8)	−0.0296 (12)
N1	0.0283 (8)	0.0306 (8)	0.0247 (8)	−0.0165 (7)	0.0083 (6)	−0.0104 (6)
N2	0.0165 (6)	0.0182 (6)	0.0206 (7)	−0.0070 (5)	0.0007 (5)	−0.0070 (5)
N3	0.0135 (6)	0.0132 (6)	0.0180 (6)	−0.0037 (5)	−0.0012 (5)	−0.0070 (5)
N4	0.0270 (7)	0.0138 (6)	0.0158 (6)	−0.0043 (5)	−0.0024 (5)	−0.0057 (5)
N5	0.0197 (6)	0.0118 (6)	0.0161 (6)	−0.0025 (5)	−0.0014 (5)	−0.0057 (5)
N6	0.0191 (6)	0.0125 (6)	0.0139 (6)	−0.0039 (5)	0.0012 (5)	−0.0057 (5)
O1	0.0235 (6)	0.0494 (9)	0.0212 (6)	−0.0089 (6)	−0.0023 (5)	−0.0151 (6)
Pd1	0.01665 (6)	0.01189 (6)	0.01366 (6)	−0.00362 (4)	−0.00014 (4)	−0.00610 (4)
S1	0.0335 (2)	0.02045 (19)	0.01642 (18)	−0.01367 (17)	0.00511 (16)	−0.00789 (15)
S2	0.02174 (18)	0.01444 (17)	0.01425 (17)	−0.00566 (14)	0.00208 (14)	−0.00749 (14)

### Geometric parameters (Å, º)

**Table:**

C1—N1	1.450 (2)	C37B—H37F	0.9800
C1—H1A	0.9800	C11—C19	1.525 (3)
C1—H1B	0.9800	C11—C20	1.532 (3)
C1—H1C	0.9800	C11—C12	1.537 (2)
C2—N2	1.304 (2)	C12—C13	1.401 (2)
C2—N1	1.350 (2)	C13—C14	1.388 (2)
C2—S1	1.7520 (17)	C13—H13A	0.9500
C3—N3	1.298 (2)	C14—C15	1.508 (2)
C3—C5	1.479 (2)	C15—H15A	0.9800
C3—C4	1.499 (2)	C15—H15B	0.9800
C4—H4A	0.9800	C15—H15C	0.9800
C4—H4B	0.9800	C17—H17A	0.9800
C4—H4C	0.9800	C17—H17B	0.9800
C5—C6	1.393 (2)	C17—H17C	0.9800
C5—C14	1.401 (2)	C19—H19A	0.9800
C6—C7	1.396 (2)	C19—H19B	0.9800
C6—H6A	0.9500	C19—H19C	0.9800
C7—C12	1.398 (2)	C20—H20A	0.9800
C7—C8	1.528 (2)	C20—H20B	0.9800
C8—C17	1.521 (3)	C20—H20C	0.9800
C8—C9A	1.524 (3)	C21—N4	1.453 (2)
C8—C16A	1.632 (4)	C21—H21A	0.9800
C8—C16B	1.331 (6)	C21—H21B	0.9800
C8—C9B	1.659 (5)	C21—H21C	0.9800
C9A—C10A	1.523 (4)	C22—N5	1.306 (2)
C9A—H9A	0.9900	C22—N4	1.344 (2)
C9A—H9B	0.9900	C22—S2	1.7644 (16)
C10A—C18A	1.527 (17)	C23—N6	1.292 (2)
C10A—C11	1.584 (4)	C23—C25	1.491 (2)
C10A—H10A	1.0000	C23—C24	1.495 (2)
C16A—H16A	0.9800	C24—H24A	0.9800
C16A—H16B	0.9800	C24—H24B	0.9800
C16A—H16C	0.9800	C24—H24C	0.9800
C18A—H18A	0.9800	C25—C26	1.388 (2)
C18A—H18B	0.9800	C25—C34	1.400 (2)
C18A—H18C	0.9800	C26—C27	1.400 (2)
C29A—C30A	1.500 (5)	C26—H26A	0.9500
C29A—C28	1.532 (4)	C27—C32	1.397 (2)
C29A—H29A	0.9900	C27—C28	1.531 (2)
C29A—H29B	0.9900	C28—C36	1.518 (3)
C30A—C38A	1.542 (17)	C31—C40	1.526 (3)
C30A—C31	1.605 (4)	C31—C39	1.529 (3)
C30A—H30A	1.0000	C31—C32	1.537 (2)
C38A—H38A	0.9800	C32—C33	1.404 (2)
C38A—H38B	0.9800	C33—C34	1.385 (2)
C38A—H38C	0.9800	C33—H33A	0.9500
C37A—C28	1.582 (5)	C34—C35	1.510 (2)
C37A—H37A	0.9800	C35—H35A	0.9800
C37A—H37B	0.9800	C35—H35B	0.9800
C37A—H37C	0.9800	C35—H35C	0.9800
C9B—C10B	1.470 (7)	C36—H36A	0.9800
C9B—H9C	0.9900	C36—H36B	0.9800
C9B—H9D	0.9900	C36—H36C	0.9800
C10B—C11	1.510 (6)	C39—H39A	0.9800
C10B—C18B	1.55 (3)	C39—H39B	0.9800
C10B—H10B	1.0000	C39—H39C	0.9800
C16B—H16D	0.9800	C40—H40A	0.9800
C16B—H16E	0.9800	C40—H40B	0.9800
C16B—H16F	0.9800	C40—H40C	0.9800
C18B—H18D	0.9800	C41—C42	1.489 (3)
C18B—H18E	0.9800	C41—H41A	0.9800
C18B—H18F	0.9800	C41—H41B	0.9800
C29B—C30B	1.529 (8)	C41—H41C	0.9800
C29B—C28	1.568 (6)	C42—O1	1.416 (3)
C29B—H29C	0.9900	C42—H42A	0.9900
C29B—H29D	0.9900	C42—H42B	0.9900
C30B—C31	1.541 (5)	N1—H1	0.8800
C30B—C38B	1.54 (3)	N2—N3	1.3970 (18)
C30B—H30B	1.0000	N3—Pd1	2.0408 (13)
C38B—H38D	0.9800	N4—H2	0.8800
C38B—H38E	0.9800	N5—N6	1.4056 (18)
C38B—H38F	0.9800	N6—Pd1	2.0209 (14)
C37B—C28	1.449 (11)	O1—H3	0.8400
C37B—H37D	0.9800	Pd1—S1	2.2893 (6)
C37B—H37E	0.9800	Pd1—S2	2.3253 (6)
			
N1—C1—H1A	109.5	C14—C13—C12	123.78 (16)
N1—C1—H1B	109.5	C14—C13—H13A	118.1
H1A—C1—H1B	109.5	C12—C13—H13A	118.1
N1—C1—H1C	109.5	C13—C14—C5	117.59 (15)
H1A—C1—H1C	109.5	C13—C14—C15	120.23 (16)
H1B—C1—H1C	109.5	C5—C14—C15	122.06 (16)
N2—C2—N1	117.80 (15)	C14—C15—H15A	109.5
N2—C2—S1	125.39 (13)	C14—C15—H15B	109.5
N1—C2—S1	116.77 (13)	H15A—C15—H15B	109.5
N3—C3—C5	118.93 (14)	C14—C15—H15C	109.5
N3—C3—C4	120.70 (15)	H15A—C15—H15C	109.5
C5—C3—C4	119.89 (14)	H15B—C15—H15C	109.5
C3—C4—H4A	109.5	C8—C17—H17A	109.5
C3—C4—H4B	109.5	C8—C17—H17B	109.5
H4A—C4—H4B	109.5	H17A—C17—H17B	109.5
C3—C4—H4C	109.5	C8—C17—H17C	109.5
H4A—C4—H4C	109.5	H17A—C17—H17C	109.5
H4B—C4—H4C	109.5	H17B—C17—H17C	109.5
C6—C5—C14	119.20 (15)	C11—C19—H19A	109.5
C6—C5—C3	117.09 (15)	C11—C19—H19B	109.5
C14—C5—C3	123.68 (15)	H19A—C19—H19B	109.5
C5—C6—C7	122.82 (16)	C11—C19—H19C	109.5
C5—C6—H6A	118.6	H19A—C19—H19C	109.5
C7—C6—H6A	118.6	H19B—C19—H19C	109.5
C6—C7—C12	118.41 (15)	C11—C20—H20A	109.5
C6—C7—C8	118.11 (16)	C11—C20—H20B	109.5
C12—C7—C8	123.48 (15)	H20A—C20—H20B	109.5
C17—C8—C9A	117.28 (19)	C11—C20—H20C	109.5
C17—C8—C7	109.92 (16)	H20A—C20—H20C	109.5
C9A—C8—C7	109.84 (18)	H20B—C20—H20C	109.5
C17—C8—C16A	105.1 (2)	N4—C21—H21A	109.5
C9A—C8—C16A	105.3 (2)	N4—C21—H21B	109.5
C7—C8—C16A	108.91 (18)	H21A—C21—H21B	109.5
C17—C8—C16B	115.3 (3)	N4—C21—H21C	109.5
C7—C8—C16B	117.0 (3)	H21A—C21—H21C	109.5
C17—C8—C9B	87.9 (2)	H21B—C21—H21C	109.5
C7—C8—C9B	104.2 (2)	N5—C22—N4	117.82 (14)
C16B—C8—C9B	118.6 (3)	N5—C22—S2	124.01 (12)
C10A—C9A—C8	110.8 (2)	N4—C22—S2	118.06 (12)
C10A—C9A—H9A	109.5	N6—C23—C25	119.97 (14)
C8—C9A—H9A	109.5	N6—C23—C24	121.73 (14)
C10A—C9A—H9B	109.5	C25—C23—C24	118.27 (14)
C8—C9A—H9B	109.5	C23—C24—H24A	109.5
H9A—C9A—H9B	108.1	C23—C24—H24B	109.5
C9A—C10A—C18A	108.4 (6)	H24A—C24—H24B	109.5
C9A—C10A—C11	110.4 (2)	C23—C24—H24C	109.5
C18A—C10A—C11	113.4 (6)	H24A—C24—H24C	109.5
C9A—C10A—H10A	108.2	H24B—C24—H24C	109.5
C18A—C10A—H10A	108.2	C26—C25—C34	119.49 (14)
C11—C10A—H10A	108.2	C26—C25—C23	118.98 (14)
C8—C16A—H16A	109.5	C34—C25—C23	121.53 (14)
C8—C16A—H16B	109.5	C25—C26—C27	122.63 (15)
H16A—C16A—H16B	109.5	C25—C26—H26A	118.7
C8—C16A—H16C	109.5	C27—C26—H26A	118.7
H16A—C16A—H16C	109.5	C32—C27—C26	118.43 (15)
H16B—C16A—H16C	109.5	C32—C27—C28	122.95 (15)
C10A—C18A—H18A	109.5	C26—C27—C28	118.62 (15)
C10A—C18A—H18B	109.5	C37B—C28—C36	120.8 (4)
H18A—C18A—H18B	109.5	C36—C28—C29A	119.0 (2)
C10A—C18A—H18C	109.5	C37B—C28—C27	110.2 (4)
H18A—C18A—H18C	109.5	C36—C28—C27	110.68 (16)
H18B—C18A—H18C	109.5	C29A—C28—C27	109.75 (18)
C30A—C29A—C28	112.7 (2)	C37B—C28—C29B	112.0 (4)
C30A—C29A—H29A	109.1	C36—C28—C29B	94.0 (3)
C28—C29A—H29A	109.1	C27—C28—C29B	107.5 (2)
C30A—C29A—H29B	109.1	C36—C28—C37A	102.8 (3)
C28—C29A—H29B	109.1	C29A—C28—C37A	103.5 (2)
H29A—C29A—H29B	107.8	C27—C28—C37A	110.5 (2)
C29A—C30A—C38A	109.8 (6)	C40—C31—C39	107.89 (17)
C29A—C30A—C31	110.9 (2)	C40—C31—C32	109.63 (15)
C38A—C30A—C31	111.4 (6)	C39—C31—C32	108.17 (14)
C29A—C30A—H30A	108.2	C40—C31—C30B	96.3 (3)
C38A—C30A—H30A	108.2	C39—C31—C30B	124.6 (3)
C31—C30A—H30A	108.2	C32—C31—C30B	109.1 (2)
C30A—C38A—H38A	109.5	C40—C31—C30A	121.1 (2)
C30A—C38A—H38B	109.5	C39—C31—C30A	98.86 (18)
H38A—C38A—H38B	109.5	C32—C31—C30A	110.07 (17)
C30A—C38A—H38C	109.5	C27—C32—C33	118.04 (15)
H38A—C38A—H38C	109.5	C27—C32—C31	123.80 (15)
H38B—C38A—H38C	109.5	C33—C32—C31	118.15 (15)
C28—C37A—H37A	109.5	C34—C33—C32	123.84 (15)
C28—C37A—H37B	109.5	C34—C33—H33A	118.1
H37A—C37A—H37B	109.5	C32—C33—H33A	118.1
C28—C37A—H37C	109.5	C33—C34—C25	117.50 (15)
H37A—C37A—H37C	109.5	C33—C34—C35	120.24 (15)
H37B—C37A—H37C	109.5	C25—C34—C35	122.25 (15)
C10B—C9B—C8	110.5 (3)	C34—C35—H35A	109.5
C10B—C9B—H9C	109.6	C34—C35—H35B	109.5
C8—C9B—H9C	109.6	H35A—C35—H35B	109.5
C10B—C9B—H9D	109.6	C34—C35—H35C	109.5
C8—C9B—H9D	109.6	H35A—C35—H35C	109.5
H9C—C9B—H9D	108.1	H35B—C35—H35C	109.5
C9B—C10B—C11	109.4 (4)	C28—C36—H36A	109.5
C9B—C10B—C18B	110.4 (10)	C28—C36—H36B	109.5
C11—C10B—C18B	114.4 (11)	H36A—C36—H36B	109.5
C9B—C10B—H10B	107.5	C28—C36—H36C	109.5
C11—C10B—H10B	107.5	H36A—C36—H36C	109.5
C18B—C10B—H10B	107.5	H36B—C36—H36C	109.5
C8—C16B—H16D	109.5	C31—C39—H39A	109.5
C8—C16B—H16E	109.5	C31—C39—H39B	109.5
H16D—C16B—H16E	109.5	H39A—C39—H39B	109.5
C8—C16B—H16F	109.5	C31—C39—H39C	109.5
H16D—C16B—H16F	109.5	H39A—C39—H39C	109.5
H16E—C16B—H16F	109.5	H39B—C39—H39C	109.5
C10B—C18B—H18D	109.5	C31—C40—H40A	109.5
C10B—C18B—H18E	109.5	C31—C40—H40B	109.5
H18D—C18B—H18E	109.5	H40A—C40—H40B	109.5
C10B—C18B—H18F	109.5	C31—C40—H40C	109.5
H18D—C18B—H18F	109.5	H40A—C40—H40C	109.5
H18E—C18B—H18F	109.5	H40B—C40—H40C	109.5
C30B—C29B—C28	112.6 (4)	C42—C41—H41A	109.5
C30B—C29B—H29C	109.1	C42—C41—H41B	109.5
C28—C29B—H29C	109.1	H41A—C41—H41B	109.5
C30B—C29B—H29D	109.1	C42—C41—H41C	109.5
C28—C29B—H29D	109.1	H41A—C41—H41C	109.5
H29C—C29B—H29D	107.8	H41B—C41—H41C	109.5
C29B—C30B—C31	106.5 (4)	O1—C42—C41	110.93 (18)
C29B—C30B—C38B	108.8 (10)	O1—C42—H42A	109.5
C31—C30B—C38B	113.5 (10)	C41—C42—H42A	109.5
C29B—C30B—H30B	109.3	O1—C42—H42B	109.5
C31—C30B—H30B	109.3	C41—C42—H42B	109.5
C38B—C30B—H30B	109.3	H42A—C42—H42B	108.0
C30B—C38B—H38D	109.5	C2—N1—C1	121.71 (16)
C30B—C38B—H38E	109.5	C2—N1—H1	119.1
H38D—C38B—H38E	109.5	C1—N1—H1	119.1
C30B—C38B—H38F	109.5	C2—N2—N3	112.94 (13)
H38D—C38B—H38F	109.5	C3—N3—N2	113.87 (13)
H38E—C38B—H38F	109.5	C3—N3—Pd1	126.67 (11)
C28—C37B—H37D	109.5	N2—N3—Pd1	119.44 (10)
C28—C37B—H37E	109.5	C22—N4—C21	119.90 (14)
H37D—C37B—H37E	109.5	C22—N4—H2	120.1
C28—C37B—H37F	109.5	C21—N4—H2	120.1
H37D—C37B—H37F	109.5	C22—N5—N6	111.48 (12)
H37E—C37B—H37F	109.5	C23—N6—N5	114.58 (13)
C10B—C11—C19	94.5 (2)	C23—N6—Pd1	130.09 (11)
C10B—C11—C20	121.4 (2)	N5—N6—Pd1	115.23 (10)
C19—C11—C20	108.82 (18)	C42—O1—H3	109.5
C10B—C11—C12	112.2 (2)	N6—Pd1—N3	178.02 (5)
C19—C11—C12	108.26 (15)	N6—Pd1—S1	97.92 (4)
C20—C11—C12	109.91 (15)	N3—Pd1—S1	82.82 (4)
C19—C11—C10A	117.58 (19)	N6—Pd1—S2	80.32 (4)
C20—C11—C10A	104.05 (18)	N3—Pd1—S2	98.47 (5)
C12—C11—C10A	108.05 (17)	S1—Pd1—S2	164.631 (17)
C7—C12—C13	118.13 (15)	C2—S1—Pd1	95.29 (6)
C7—C12—C11	123.21 (16)	C22—S2—Pd1	91.28 (6)
C13—C12—C11	118.60 (16)		
			
N3—C3—C5—C6	−54.0 (2)	C30A—C29A—C28—C36	78.1 (3)
C4—C3—C5—C6	118.21 (17)	C30A—C29A—C28—C27	−50.8 (3)
N3—C3—C5—C14	128.43 (17)	C30A—C29A—C28—C37A	−168.8 (3)
C4—C3—C5—C14	−59.4 (2)	C32—C27—C28—C37B	107.5 (4)
C14—C5—C6—C7	−1.4 (2)	C26—C27—C28—C37B	−72.3 (4)
C3—C5—C6—C7	−179.08 (14)	C32—C27—C28—C36	−116.2 (2)
C5—C6—C7—C12	−1.1 (2)	C26—C27—C28—C36	63.9 (2)
C5—C6—C7—C8	179.08 (15)	C32—C27—C28—C29A	17.2 (3)
C6—C7—C8—C17	−68.7 (2)	C26—C27—C28—C29A	−162.69 (19)
C12—C7—C8—C17	111.49 (19)	C32—C27—C28—C29B	−14.8 (3)
C6—C7—C8—C9A	160.86 (17)	C26—C27—C28—C29B	165.3 (3)
C12—C7—C8—C9A	−19.0 (3)	C32—C27—C28—C37A	130.6 (3)
C6—C7—C8—C16A	46.0 (2)	C26—C27—C28—C37A	−49.2 (3)
C12—C7—C8—C16A	−133.9 (2)	C30B—C29B—C28—C37B	−71.8 (6)
C6—C7—C8—C16B	65.3 (4)	C30B—C29B—C28—C36	162.6 (4)
C12—C7—C8—C16B	−114.5 (3)	C30B—C29B—C28—C27	49.5 (5)
C6—C7—C8—C9B	−161.6 (2)	C29B—C30B—C31—C40	166.5 (4)
C12—C7—C8—C9B	18.6 (3)	C38B—C30B—C31—C40	−73.8 (10)
C17—C8—C9A—C10A	−76.7 (3)	C29B—C30B—C31—C39	−76.6 (4)
C7—C8—C9A—C10A	49.7 (3)	C38B—C30B—C31—C39	43.1 (10)
C16A—C8—C9A—C10A	166.8 (2)	C29B—C30B—C31—C32	53.2 (4)
C8—C9A—C10A—C18A	166.9 (7)	C38B—C30B—C31—C32	172.9 (10)
C8—C9A—C10A—C11	−68.3 (3)	C29A—C30A—C31—C40	88.6 (3)
C28—C29A—C30A—C38A	−171.8 (5)	C38A—C30A—C31—C40	−34.0 (6)
C28—C29A—C30A—C31	64.7 (3)	C29A—C30A—C31—C39	−154.2 (2)
C17—C8—C9B—C10B	−166.3 (4)	C38A—C30A—C31—C39	83.2 (5)
C7—C8—C9B—C10B	−56.3 (4)	C29A—C30A—C31—C32	−41.1 (3)
C16B—C8—C9B—C10B	75.9 (5)	C38A—C30A—C31—C32	−163.7 (5)
C8—C9B—C10B—C11	71.0 (4)	C26—C27—C32—C33	1.6 (2)
C8—C9B—C10B—C18B	−162.2 (12)	C28—C27—C32—C33	−178.33 (15)
C28—C29B—C30B—C31	−71.5 (5)	C26—C27—C32—C31	−177.59 (15)
C28—C29B—C30B—C38B	165.8 (10)	C28—C27—C32—C31	2.5 (2)
C9B—C10B—C11—C19	−155.0 (3)	C40—C31—C32—C27	−126.5 (2)
C18B—C10B—C11—C19	80.6 (12)	C39—C31—C32—C27	116.09 (19)
C9B—C10B—C11—C20	89.7 (4)	C30B—C31—C32—C27	−22.2 (3)
C18B—C10B—C11—C20	−34.7 (12)	C30A—C31—C32—C27	9.1 (2)
C9B—C10B—C11—C12	−43.2 (4)	C40—C31—C32—C33	54.3 (2)
C18B—C10B—C11—C12	−167.6 (11)	C39—C31—C32—C33	−63.1 (2)
C9A—C10A—C11—C19	−73.1 (3)	C30B—C31—C32—C33	158.6 (3)
C18A—C10A—C11—C19	48.7 (7)	C30A—C31—C32—C33	−170.06 (18)
C9A—C10A—C11—C20	166.5 (2)	C27—C32—C33—C34	−1.3 (2)
C18A—C10A—C11—C20	−71.6 (7)	C31—C32—C33—C34	177.92 (15)
C9A—C10A—C11—C12	49.7 (2)	C32—C33—C34—C25	−0.8 (2)
C18A—C10A—C11—C12	171.6 (7)	C32—C33—C34—C35	178.11 (16)
C6—C7—C12—C13	2.3 (2)	C26—C25—C34—C33	2.6 (2)
C8—C7—C12—C13	−177.87 (16)	C23—C25—C34—C33	−177.14 (15)
C6—C7—C12—C11	−174.95 (15)	C26—C25—C34—C35	−176.33 (16)
C8—C7—C12—C11	4.9 (2)	C23—C25—C34—C35	4.0 (2)
C10B—C11—C12—C7	5.8 (3)	N2—C2—N1—C1	−10.2 (3)
C19—C11—C12—C7	108.7 (2)	S1—C2—N1—C1	167.84 (15)
C20—C11—C12—C7	−132.54 (18)	N1—C2—N2—N3	172.20 (14)
C10A—C11—C12—C7	−19.6 (2)	S1—C2—N2—N3	−5.6 (2)
C10B—C11—C12—C13	−171.5 (2)	C5—C3—N3—N2	167.56 (13)
C19—C11—C12—C13	−68.5 (2)	C4—C3—N3—N2	−4.5 (2)
C20—C11—C12—C13	50.2 (2)	C5—C3—N3—Pd1	−14.3 (2)
C10A—C11—C12—C13	163.15 (17)	C4—C3—N3—Pd1	173.55 (12)
C7—C12—C13—C14	−1.2 (2)	C2—N2—N3—C3	−161.72 (14)
C11—C12—C13—C14	176.18 (15)	C2—N2—N3—Pd1	20.03 (17)
C12—C13—C14—C5	−1.2 (2)	N5—C22—N4—C21	−2.3 (2)
C12—C13—C14—C15	−177.37 (15)	S2—C22—N4—C21	174.14 (12)
C6—C5—C14—C13	2.5 (2)	N4—C22—N5—N6	174.48 (13)
C3—C5—C14—C13	180.00 (14)	S2—C22—N5—N6	−1.68 (19)
C6—C5—C14—C15	178.52 (15)	C25—C23—N6—N5	3.4 (2)
C3—C5—C14—C15	−3.9 (2)	C24—C23—N6—N5	−178.64 (14)
N6—C23—C25—C26	−110.66 (18)	C25—C23—N6—Pd1	−172.87 (11)
C24—C23—C25—C26	71.3 (2)	C24—C23—N6—Pd1	5.1 (2)
N6—C23—C25—C34	69.1 (2)	C22—N5—N6—C23	−142.16 (14)
C24—C23—C25—C34	−108.96 (18)	C22—N5—N6—Pd1	34.70 (16)
C34—C25—C26—C27	−2.3 (2)	N2—C2—S1—Pd1	−8.19 (15)
C23—C25—C26—C27	177.37 (14)	N1—C2—S1—Pd1	173.96 (13)
C25—C26—C27—C32	0.2 (2)	N5—C22—S2—Pd1	−24.45 (14)
C25—C26—C27—C28	−179.90 (15)	N4—C22—S2—Pd1	159.40 (12)

### Hydrogen-bond geometry (Å, º)

**Table:**

*D*—H···*A*	*D*—H	H···*A*	*D*···*A*	*D*—H···*A*
C24—H24*C*···S1	0.98	2.73	3.5061 (19)	136
N1—H1···S1^i^	0.88	2.57	3.411 (2)	160
N4—H2···O1	0.88	2.01	2.879 (2)	167
O1—H3···S2^ii^	0.84	2.43	3.2596 (16)	169

Symmetry codes: (i) −*x*+2, −*y*+2, −*z*+1; (ii) −*x*+2, −*y*+1, −*z*+2.
